# Loss of PADI2 and PADI4 ameliorates sepsis-induced acute lung injury by suppressing NLRP3^+^ macrophages

**DOI:** 10.1172/jci.insight.181686

**Published:** 2024-11-22

**Authors:** Xin Yu, Yujing Song, Tao Dong, Wenlu Ouyang, Liujiazi Shao, Chao Quan, Kyung Eun Lee, Tao Tan, Allan Tsung, Katsuo Kurabayashi, Hasan B. Alam, Mao Zhang, Jianjie Ma, Yongqing Li

**Affiliations:** 1 Department of Surgery, University of Michigan Health System, Ann Arbor, Michigan, USA.; 2 Department of Emergency Medicine, Second Affiliated Hospital, Zhejiang University School of Medicine, Hangzhou, Zhejiang, China.; 3 Department of Mechanical and Aerospace Engineering, New York University, Brooklyn, New York, USA.; 4 Department of Mechanical Engineering, University of Michigan, Ann Arbor, Michigan, USA.; 5 Department of Physiology, Xuzhou Medical University, Xu Zhou, Jiangsu, China.; 6 Department of Metabolism and Endocrinology, The Second Xiangya Hospital, Changsha, China.; 7 Department of Anesthesiology, Beijing Friendship Hospital, Capital Medical University, Xicheng District, Beijing, China.; 8 Department of Urology, The Xiangya Hospital, Changsha, China.; 9 Department of Surgery, Division of Surgical Science, University of Virginia, Charlottesville, Virginia, USA.; 10 Department of Chemical and Biomolecular Engineering, New York University, Brooklyn, New York, USA.; 11 Department of Surgery, Northwestern University, Arkes Pavilion, Chicago, Illinois, USA.

**Keywords:** Immunology, Inflammation, Bacterial infections, Macrophages, Surgery

## Abstract

Sepsis-induced acute lung injury (ALI) is prevalent in patients with sepsis and has a high mortality rate. Peptidyl arginine deiminase 2 (PADI2) and PADI4 play crucial roles in mediating the host’s immune response in sepsis, but their specific functions remain unclear. Our study shows that *Padi2^–/–^ Padi4^–/–^* double KO (DKO) improved survival, reduced lung injury, and decreased bacterial load in *Pseudomonas aeruginosa* (PA) pneumonia–induced sepsis mice. Using single-cell RNA-Seq (scRNA-Seq), we found that the deletion of *Padi2* and *Padi4* reduced the *Nlrp3*^+^ proinflammatory macrophages and fostered *Chil3*^+^ myeloid cell differentiation into antiinflammatory macrophages. Additionally, we observed the regulatory role of the NLRP3/Ym1 axis upon DKO, confirmed by *Chil3* knockdown and *Nlrp3*-KO experiments. Thus, eliminating *Padi2* and *Padi4* enhanced the polarization of Ym1^+^ M2 macrophages by suppressing NLRP3, aiding in inflammation resolution and lung tissue repair. This study unveils the PADIs/NLRP3/Ym1 pathway as a potential target in treatment of sepsis-induced ALI.

## Abstract 2

Yu *et al*. demonstrate that <a>*Padi2*/*Padi4* double knockout (DKO) in mice boosts survival, mitigates acute lung injury (ALI), and lessens bacterial load in sepsis. Their single-cell RNA-sequencing study highlights the DKO’s role in reducing pro-inflammatory macrophages and promoting anti-inflammatory responses, suggesting the PADI2/PADI4-NLRP3-Ym1 pathway as a therapeutic target in sepsis-induced ALI.</a>

## Graphical abstract

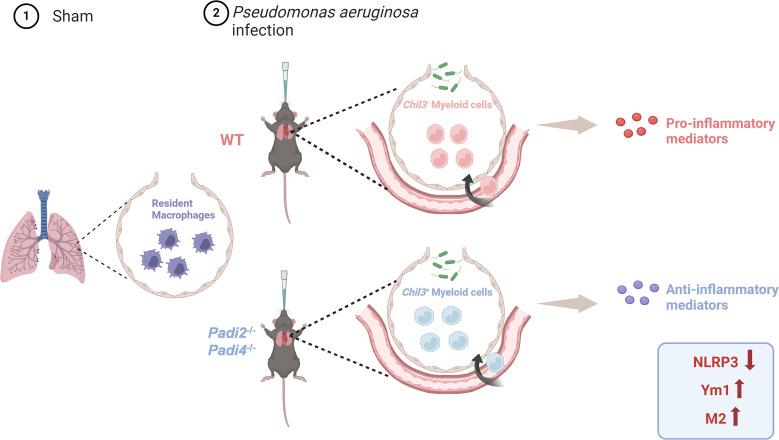

## Introduction

Sepsis, a life-threatening condition characterized by organ dysfunction due to a dysregulated host response to infection, has high morbidity and mortality rates (1, 2). It induces severe pulmonary inflammation, leading to acute lung injury (ALI) and causing irreversible lung damage (3). An international study of 11,000 severe sepsis cases showed that 57% were associated with gram-negative bacteria infections, with the lung being the primary infection site in 47% of these patients (4). *Pseudomonas aeruginosa* (PA), a prominent gram-negative bacterium, is a major contributor to hospital-acquired infections, especially pneumonia in an intensive care unit (5), which raises the risk of sepsis and mortality compared with other pneumonia-causing pathogens (6). Considering ALI’s close link to sepsis, we used a PA pneumonia–induced sepsis mouse model to investigate alveolar microenvironment alterations and lung injury after sepsis (7).

Peptidyl arginine deiminases (PADIs, also known as PADs) are a family of enzymes that play a critical role in citrullination, affecting protein function and various physiological processes and diseases (8). Among the 5 related calcium-dependent PADI isoforms, PADI2 and PADI4 are highly expressed in monocytes and macrophages (9, 10), and they play substantial roles in the immune response to sepsis (11–13). Elevated levels of PADI4 have been linked to increased ICU mortality in patients with septic shock (14). Higher concentrations of PADI2 have been detected in serum and BALF of patients with sepsis and septic mice (15). Targeting these enzymes with a PADI2/PADI4 inhibitor, YW3-56, has shown improved survival and reduced lung injury in septic mice (16). Deletion of *Padi2* enhances survival and diminishes lung injury in sepsis (7, 15), while *Padi4* deficiency benefits the LPS-induced endotoxic shock model but not in cecal ligation and puncture (CLP) or pneumonia models (17, 18).

While research has predominantly focused on the individual deletion of *Padi2* or *Padi4*, the combined effect of *Padi2* and *Padi4* double KO (DKO; *Padi2^–/–^ Padi4^–/–^*) on sepsis and its progression, particularly in the context of lung injury, is not well understood. Considering the potential critical roles of both PADI2 and PADI4 in sepsis, we hypothesize that the deletion of both *Padi2* and *Padi4* could offer a marked therapeutic advantage. Our study aimed to fill this gap by evaluating the DKO mouse model in PA pneumonia–induced sepsis, focusing on its effect on the alveolar immune landscape and ALI development. Here, we employed single-cell RNA-Seq (scRNA-Seq) technology to map immune cell populations in bronchoalveolar lavage fluid (BALF) (19–21), elucidating the alveolar immune landscape and deciphering the intricate signaling pathways activated following infection, showing that the absence of *Padi2* and *Padi4* alters immune responses in sepsis.

## Results

### Padi2 and Padi4 deficiency reduces ALI in a PA pneumonia–induced sepsis mouse model.

**Figure 1:**
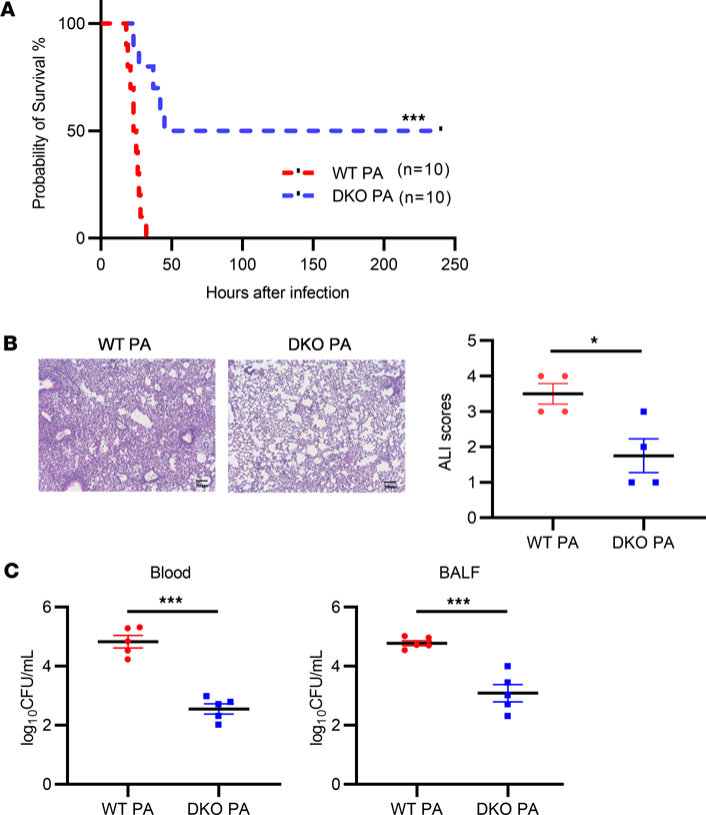
Protective effect of *Padi2* and *Padi4* deficiency against acute lung injury in a PA pneumonia–induced sepsis mouse model. (**A**) Kaplan-Meier survival rate of WT and DKO mice following intranasal inoculation with *Pseudomonas aeruginosa* (PA) at a dose of 2.5 × 10^6^ CFU per mouse. Survival was monitored for a period of 10 days after inoculation (*n* = 10 mice/group). Values are expressed as a survival percentage. (**B**) Histopathological examination of lung injury. The left panel presents H&E-stained lung tissue sections from WT and DKO mice 24 hours after PA inoculation (*n* = 4–5 mice/group). The right panel shows quantified acute lung injury (ALI) scores. Scale bars: 100 μm. (**C**) Bacterial loads measured in the blood and BALF of WT and DKO mice 24 hours after PA inoculation (*n* = 5 mice/group). Data from **A** were analyzed using log rank tests. Data from **B** and **C** were analyzed using unpaired Student’s *t* tests. Results are presented as means ± SEM. *P < 0.05; ***P <.001.

In the exploration of the effects of *Padi2* and *Padi4* DKO on ALI and survival in a PA pneumonia–induced sepsis model, we first constructed the model and validated the knockout efficiency (Supplemental Figure 1; supplemental material available online with this article; https://doi.org/10.1172/jci.insight.181686DS1). Survival of mice after PA inoculation was then monitored over a 10-day period. Remarkably, about 50% of DKO mice (*n* = 10) survived the entirety of the observation period, whereas the WT counterparts (*n* = 10) succumbed approximately 48 hours after inoculation (Figure 1A). Focusing on the lung as the primary site of infection, we next explored both functional and histopathological changes. We quantified total protein concentrations in BALF as an indicator of lung permeability, a critical parameter reflecting the integrity of the alveolar-capillary barrier compromised by PA infection. DKO mice exhibited reduced protein leakage into the alveoli compared with WT mice (Supplemental Figure 2).

The H&E staining of lung tissues was conducted 24 hours after inoculation. The analysis, performed by a pathologist blinded to the experiment, showed that DKO mice exhibited less ALI compared with WT mice, as demonstrated by reduced inflammatory cell infiltration, pulmonary edema, and alveolar hemorrhage (Figure 1B). Furthermore, to assess the effect of *Padi2* and *Padi4* deficiency on bacterial clearance, we measured bacterial loads in BALF and blood 24 hours after PA inoculation. Our analyses show reduced bacterial levels in both BALF and blood samples of DKO mice compared with WT mice, as illustrated in Figure 1C.

### Identification of 10 distinct immune cell subpopulations in BALF using scRNA-Seq in a PA-induced ALI.

**Figure 2:**
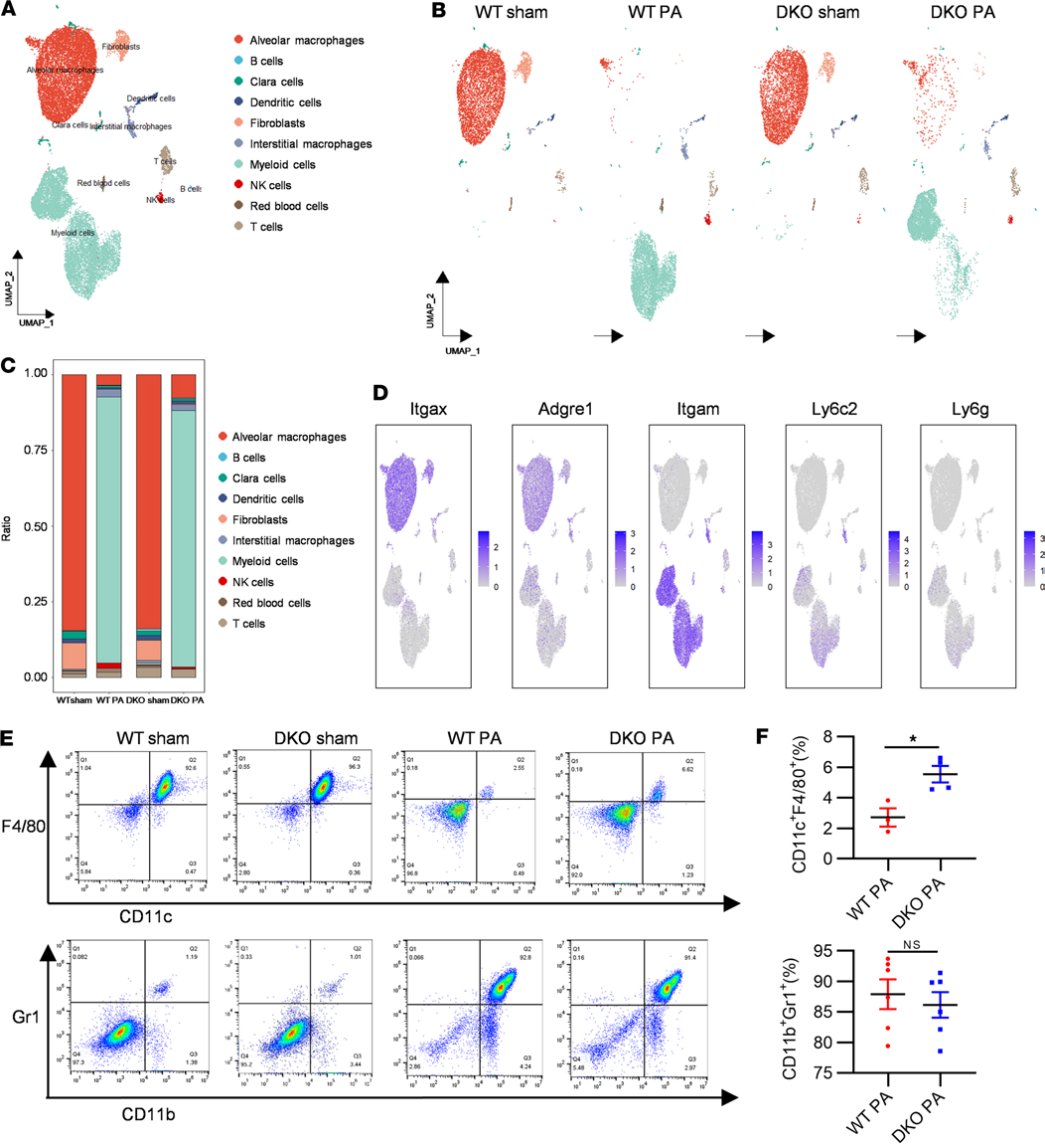
Identification of distinct immune cell subpopulations in BALF via scRNA-Seq in PA pneumonia–induced sepsis model. (**A**) Nonlinear dimensionality reduction Uniform Manifold Approximation and Projection (UMAP) analysis of 22,917 BALF cells from WT and DKO mice reveals 10 distinct clusters following unsupervised clustering. Each dot represents an individual cell, with coloring indicating cluster assignment. BALF cells from 3 mice were mixed as 1 sample for each group, including WT-sham, WT-PA, DKO-sham, and DKO-PA groups. (**B**) Experimental group–based UMAP visualization of WT-sham, WT-PA, DKO-sham, and DKO-PA. This visualization highlights the distinct immune landscapes present in the 2 mouse models, both with and without PA infection (*n* = 3 mice/group). (**C**) Distribution of cell subtype proportions among all cell populations in each experimental group (*n* = 3 mice/group). (**D**) UMAP gene expression patterns for key immune markers — *Adgre1* (F4/80) and *Itgax* (CD11c) for resident macrophages and *Ly6c2* (Ly6C), *Ly6g* (Ly6G), and *Itgam* (CD11b) for recruited myeloid cells. Regions with purple shading denote higher expression levels of these markers. (**E**) scRNA-Seq results validated by flow cytometry. The upper panel focuses on resident macrophages, identified by F4/80^+^CD11c^+^, and the lower panel on recruited myeloid cells, identified by Gr1^+^CD11b^+^ (Gr1 including 2 isoforms of Ly6C and Ly6G). (**F**) Cell population difference between the WT-PA and DKO-PA by flow cytometry (*n* = 3–6 mice/group). Results are representative of 3 independent experiments. Data were analyzed using unpaired Student’s *t* tests and are presented as mean ± SEM. *P < 0.05.

Employing 10× Genomics scRNA-Seq platform, we characterized 22,917 BALF cells from WT and DKO mice in PA-induced ALI and sham conditions. A diverse immune cell landscape was mapped via nonlinear dimensionality reduction on Uniform Manifold Approximation and Projection (UMAP) plot for WT and DKO group after integration analysis. As a result, 10 distinct clusters of BALF cells were identified by the Seurat clustering algorithm (Figure 2A and Supplemental Figure 3A). Cell type identification within each cluster was determined based on the expression of well-characterized marker genes (Supplemental Figure 3B). Figure 2B shows the UMAP visualization of cell populations from both WT and DKO mice, revealing mature alveolar macrophages (AMs) as predominant in the sham condition, while immature myeloid cells became the major population following PA inoculation in both WT and DKO mice. Additional cell populations identified included fibroblasts, Clara cells, DCs, T cells, interstitial macrophages (IMs), NK cells, B cells, and RBCs. The relative abundance of each cell type was quantified in Figure 2C. A clear cell type transition from AMs to myeloid cells was observed after PA infection compared with the sham group.

Surface markers CD11b and CD11c serve as distinguishing features between resident AMs (CD11c^hi^/CD11b^lo^) and recruited myeloid cells (CD11c^lo^/CD11b^hi^) (22). Additionally, myeloid cells could undergo further differentiation into monocytes/macrophages (Ly6C^+^Ly6G^–^) or neutrophils (Ly6G^+^Ly6C^–^) (23). To study this transition, we analyzed marker genes *Itgax* (gene name of CD11c) and *Adgre1*(gene name of F4/80) for resident AMs, alongside *Itgam* (gene name of CD11b), *Ly6c2* (gene name of Ly6C2), and *Ly6g* (gene name of Ly6G) for recruited myeloid cells. Figure 2D illustrates the relative RNA expression levels of these markers across all identified clusters. High expression levels mark the resident AM clusters as *Itgax*^+^*Adgre1*^+^ were predominantly observed in the sham condition. Following PA infection, a new cluster expressing *Itgam^+^Ly6c2^+^Ly6g^+^* begins to emerge, representing newly recruited myeloid-derived cells, despite the relatively moderate expression of *Ly6c2* and *Ly6g* genes.

To validate the observed shifts in cell populations identified through scRNA-Seq, we employed flow cytometry with cell-specific markers, including CD11c, CD11b, F4/80, and Gr1 (encompassing Ly6C and Ly6G), to stain BALF cells from both WT and DKO mice. This analysis further confirmed that AMs (F4/80^+^CD11c^+^Gr1^–^CD11b^–^), which are mature and resident within the alveolar niche, decreased in number in response to bacterial invasion. This reduction underscores the necessity for the recruitment of myeloid cells (Gr1^+^CD11b^+^F4/80^–^CD11c^–^) from the bone marrow into the bloodstream and the migration into the alveoli, where they become the predominant population (Figure 2E). Notably, the absence of *Padi2* and *Padi4* genes resulted in a higher population of resident macrophages (F4/80^+^CD11c^+^Gr1^–^CD11b^–^) within the alveoli of DKO mice compared with WT mice (Figure 2F). However, no significant differences were observed in the myeloid cell populations (Gr1^+^CD11b^+^F4/80^–^CD11c^–^) between WT and DKO groups (Figure 2F).

### Identification of a unique proinflammatory resident macrophage population (Cluster12 Nlrp3^hi^) reduced by Padi2 and Padi4 deficiency in acute inflammatory phase.

**Figure 3:**
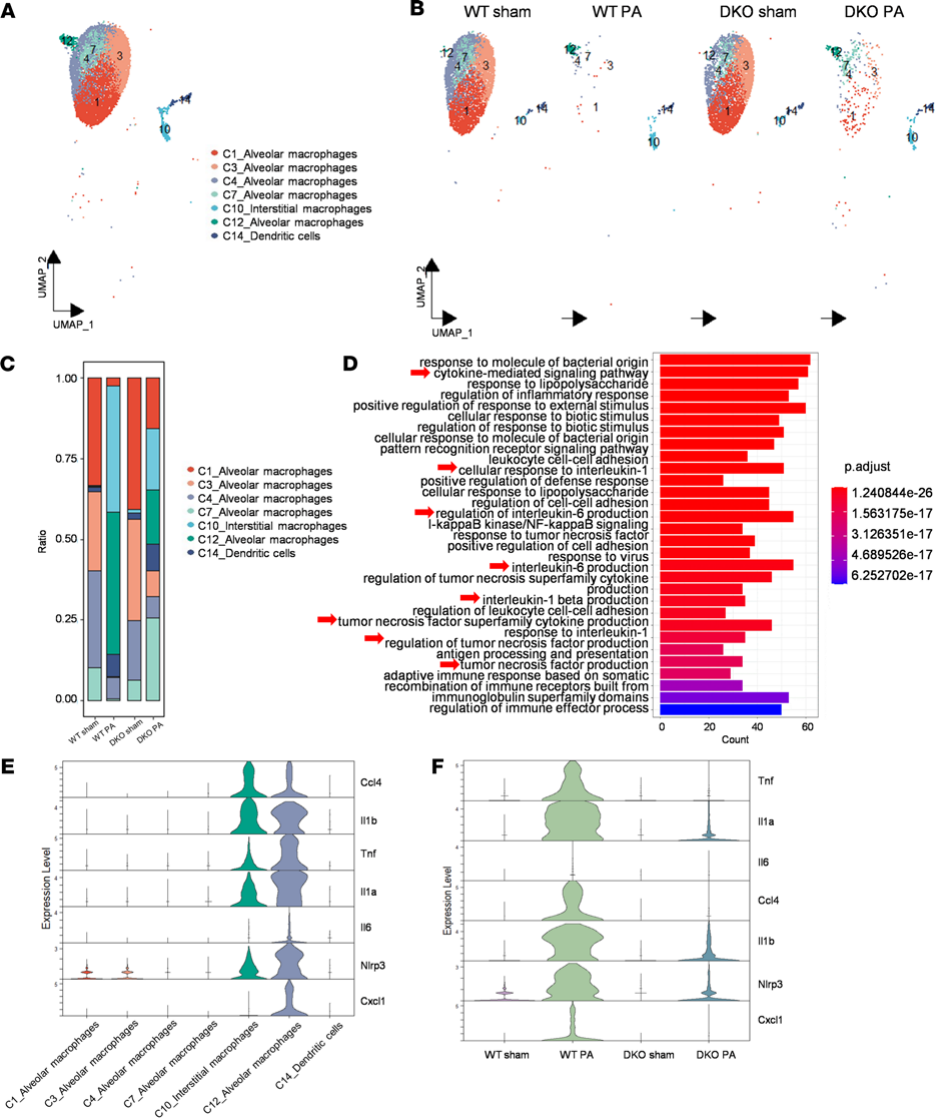
Discovery of a unique proinflammatory macrophage population (C12) reduced by *Padi2* and *Padi4* deficiency. (**A**) UMAP analysis of 7 distinct subclusters (C1, C3, C4, C7, C10, C12, and C14) of macrophages and DCs from WT and DKO mice across sham and PA conditions, with each cluster color-coded for identification (*n* = 3 mice/group). (**B**) Experimental group based UMAP visualization of macrophages and DCs from WT Sham, WT PA, DKO Sham, and DKO PA groups (*n* = 3 mice/group). (**C**) Distribution of cell subtype proportions among macrophage/DC populations in each experimental group (*n* = 3 mice/group). (**D**) Enrichment analysis of representative Gene Ontology (GO) biological pathways for C12 alveolar macrophages (AMs). (**E**) Expression profiles of proinflammatory genes (*Ccl4*, *Il1b*, *Tnf*, *Il1a*, *Il6*, *Nlrp3*, and *Cxcl1*) across macrophage/DC clusters, illustrated by violin plots. (**F**) Comparative expression of proinflammatory genes (*Tnf*, *Il1a*, *Il6*, *Ccl4*, *Il1b*, *Nlrp3*, and *Cxcl1*) in macrophages/DCs from WT and DKO mice under sham and PA groups, illustrated by violin plots (*n* = 3 mice/group).

In response to PA-induced ALI, scRNA-Seq analysis reveals 7 transcriptionally distinct subpopulations of macrophages/DCs (Figure 3A), comprising 5 AM clusters (Cluster 1 [C1], C3, C4, C7, and C12), 1 IM cluster (C10), and 1 DC cluster (C14). A heterogeneous cell distribution was observed among WT and DKO groups across these subsets, with and without PA infection at 24 hours (Figure 3, B and C). In the sham state, resident mononuclear phagocytes, including Clusters 1, 3, 4, and 7 of AMs and C14 DCs, were identified as predominant. These cells are hyporesponsive to bacteria with little or no expression of proinflammatory cytokine (*Tnf*, *Il1a*, *Il1b*, and *Il6*) and chemokine (*Cxcl1* and *Ccl4*) genes (24, 25) (Figure 3E). Following PA-induced ALI, a dramatic reduction in these clusters was observed (98.1% in WT Sham versus 10.1% in WT PA).

Contrastingly, C12 AMs emerged as a marked population post-sepsis (0.1% in WT Sham versus 43.7% in WT PA), characterized by a distinct proinflammatory signature with elevated expression of the inflammasome *Nlrp3* gene, alongside increased levels of proinflammatory cytokine and chemokine genes (Figure 3E). Gene enrichment analysis further supported the proinflammatory function of C12 *Nlrp3*^hi^ AMs, showing their involvement in cytokine production regulation and the positive regulation of inflammatory responses (Figure 3D). In contrast, other resident mononuclear phagocytes (C1, C3, C4, C7 AMs) exhibited low expression of the *Nlrp3* gene. The deletion of *Padi2* and *Padi4* resulted in a marked reduction of the C12 *Nlrp3*^hi^ AMs (43.7% in WT PA versus 16.8% in DKO PA). Concurrently, C10 IMs also increased after sepsis (0.4% in WT Sham versus 39.1% in WT PA), with a subsequent decreased level observed following *Padi2* and *Padi4* deficiency (39.1% in WT PA versus 18.7% in DKO PA). Importantly, *Padi2* and *Padi4* deficiency led to a marked decrease in the expression of *Nlrp3* and associated proinflammatory mediators (Figure 3F).

### Influences of Padi2 and Padi4 deficiency on Chil3^+^ myeloid cell differentiation into macrophages.

After PA inoculation, scRNA-Seq revealed an emergence of recruited myeloid cells as the dominant population in the alveoli, likely activated by proinflammatory cytokines and chemokines from *Nlrp3*^hi^ AMs and IMs. Given the high plasticity and crucial immunoregulatory roles of myeloid cells (26, 27), we conducted a subcluster analysis to explore how *Padi2* and *Padi4* deletions influence their immunoregulatory functions.

**Figure 4:**
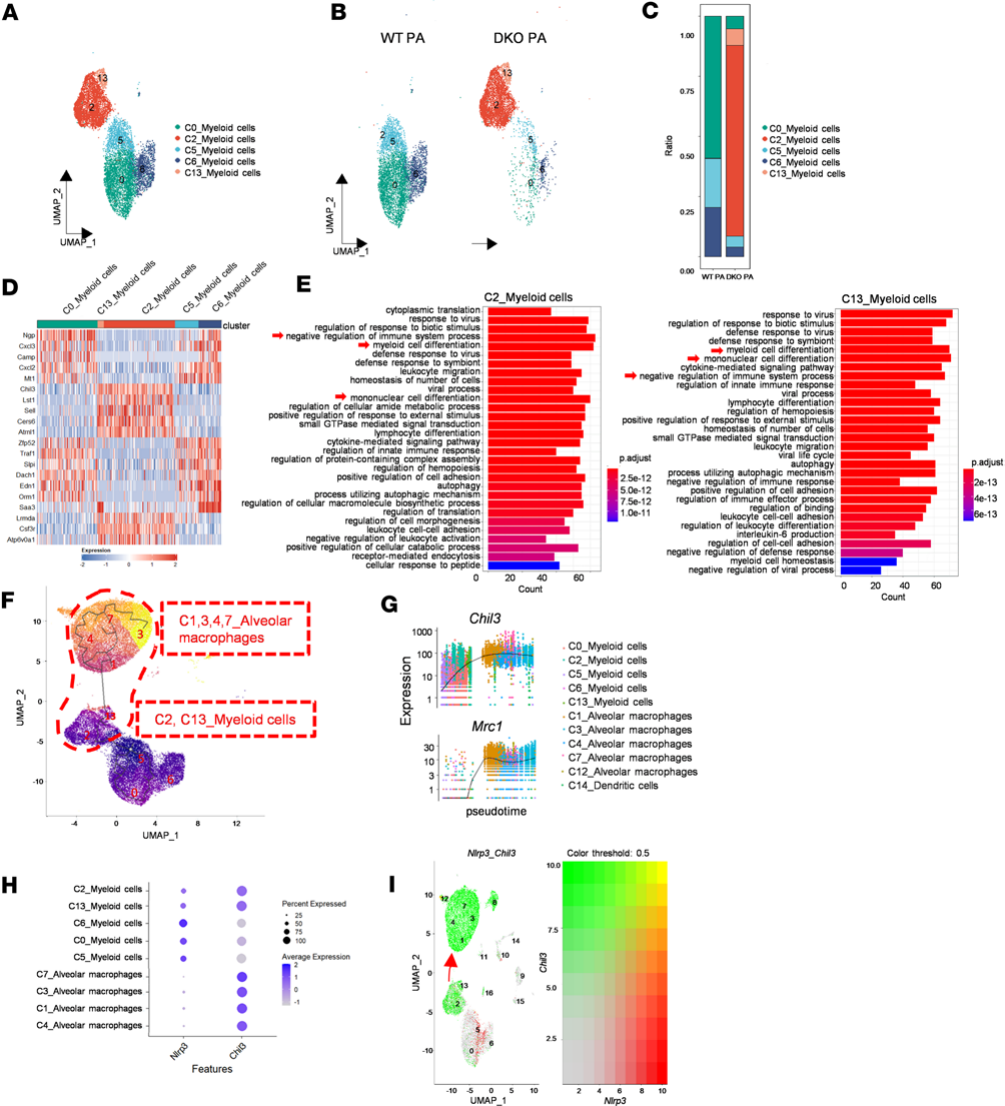
*Padi2* and *Padi4* deficiency favors the differentiation of *Chil3*^+^ myeloid cells toward macrophages. (**A**) UMAP analysis of 5 subclusters (C0, C2, C5, C6, C13) of 10,364 myeloid cells from WT and DKO mice in PA-induced sepsis model, with each cluster color-coded for identification (*n* = 3 mice/group). (**B**) Experimental group–based UMAP visualization of myeloid cells from WT PA and DKO PA groups (*n* = 3 mice/group). (**C**) Distribution of cell subtype proportions among myeloid cell populations in WT PA and DKO PA groups (*n* = 3 mice/group). (**D**) Heatmap representation of gene expression within the myeloid cell compartment from WT and DKO mice in PA-induced sepsis condition, annotated by cluster types. (**E**) Enrichment analysis of representative GO biological pathways for C2 and C13 myeloid cells. (**F**) Developmental trajectory and pseudotime reconstruction of all myeloid-derived cells, including macrophages, myeloid cells, and DCs, as inferred by Monocle 3, provide insights into cell differentiation pathways. (**G**) Pseudotime plot illustrating the expression of *Chil3* and *Mrc1* genes across all myeloid-derived cell populations, mapping changes in gene expression over pseudotime. (**H**) Coexpression patterns of *Nlrp3* and *Chil3* genes across distinct cell cluster populations, illustrated in a dot plot for a comparative overview. (**I**) Expression visualization of *Nlrp3* and *Chil3* genes across all cluster populations. Heatmap represents the relative gene expression levels.

This analysis identified 5 subclusters (C0, C2,C5, C6, and C13) of *Gr1^+^CD11b^+^* myeloid cells (Figure 4A), with distinct distributions between WT and DKO mice after PA infection (Figure 4, B and C). Notably, marked reduction of Clusters 0, 5, and 6 were observed in DKO mice compared with WT mice after sepsis (99.9% in WT PA versus 13.7% in DKO PA), alongside a dramatic increase in Clusters 2 and 13 (86.26% in DKO PA), which predominantly express *Chil3* (chitinase-like 3, gene name of Ym1), a marker generally associated with alternatively activated (M2) macrophages (28).

Further gene expression analysis between *Chil3*^–^ (Clusters 0, 5, 6) and *Chil3*^+^ (Clusters 2, 13) myeloid cells unveiled a differentiation trend influenced by *Padi2* and *Padi4* deletion (Figure 4D). C0 myeloid cells were characterized by high gene expression of *Ngp* (neutrophilic granule protein), *Camp* (cathelicidin antimicrobial peptide), *Cxcl2*, and *Cxcl3*, indicating a potential differentiation path toward neutrophil-like cells (29, 30). Clusters 5 and 6 also displayed similar gene expression patterns, with C6 notably expressing high levels of *Saa3* (serum amyloid A3), a gene involved in the cellular response to proinflammatory conditions (31). Conversely, Clusters 2 and 13 exhibited elevated expressions of *Chil3* and *Lst1* (leukocyte specific transcript 1), indicative of differentiation toward monocyte/macrophage lineage (32). Gene enrichment analysis of C2 and C13 myeloid cells also revealed their differentiation toward mononuclear cells, and the negative regulation of immune responses (Figure 4E).

Utilizing pseudotime reconstruction and developmental trajectory analysis (Figure 4F), we uncovered that, in the absence of *Padi2* and *Padi4*, *Chil3*^–^ myeloid cells (C5) exhibited a pronounced tendency to differentiate toward *Chil3^+^* cells in C2 and C13, which subsequently matured into *Chil3*^hi^ AMs (C1,3,4,7). This differentiation path was characterized by a sequential upregulation of *Chil3* and *Mrc1* (mannose receptor C type 1, gene name of CD206) markers indicative of an M2 macrophage phenotype (33), following this pseudotime order (Figure 4G). Conversely, in WT mice, where *Padi2* and *Padi4* are expressed, *Chil3*^–^ myeloid cells (C5) were more inclined to adopt a differentiation pathway toward C6 and C0, which are typified by neutrophil-like features.

Observations of *Nlrp3*^hi^ AMs and IMs secreting proinflammatory cytokines, characteristic of classically activated (M1) macrophages (34, 35), prompted us to explore the role of NLRP3 in myeloid cell differentiation after sepsis. Analyzing *Nlrp3* and *Chil3* gene expression across myeloid cells and macrophage populations (Figure 4H) revealed distinct patterns: in the sham state, resident AMs (C1,3,4,7), devoid of *Nlrp3* expression, exhibited high *Chil3* levels. After PA infection, however, *Nlrp3* expression increased in C0, C5, and C6 myeloid cells, without concurrent *Chil3* activation, unlike in C2 and C13 cells, where *Chil3* was upregulated despite low *Nlrp3* levels. Pseudotime analysis after *Padi2* and *Padi4* deletion underscored a differentiation shift in *Nlrp3^lo^Chil3^+^* myeloid cells (C2, C13) toward *Nlrp3*^–^*Chil3*^hi^ AMs (C1, C3, C4, C7) (Figure 4, F and I). Expression visualization of *Nlrp3* and *Chil3* genes across cluster populations further demonstrated *Nlrp3* and *Chil*3′s opposing expression trends across differentiation stages (Figure 4I).

### Resolution of inflammation through Chil3^+^ myeloid cell differentiation into M2 macrophages.

**Figure 5:**
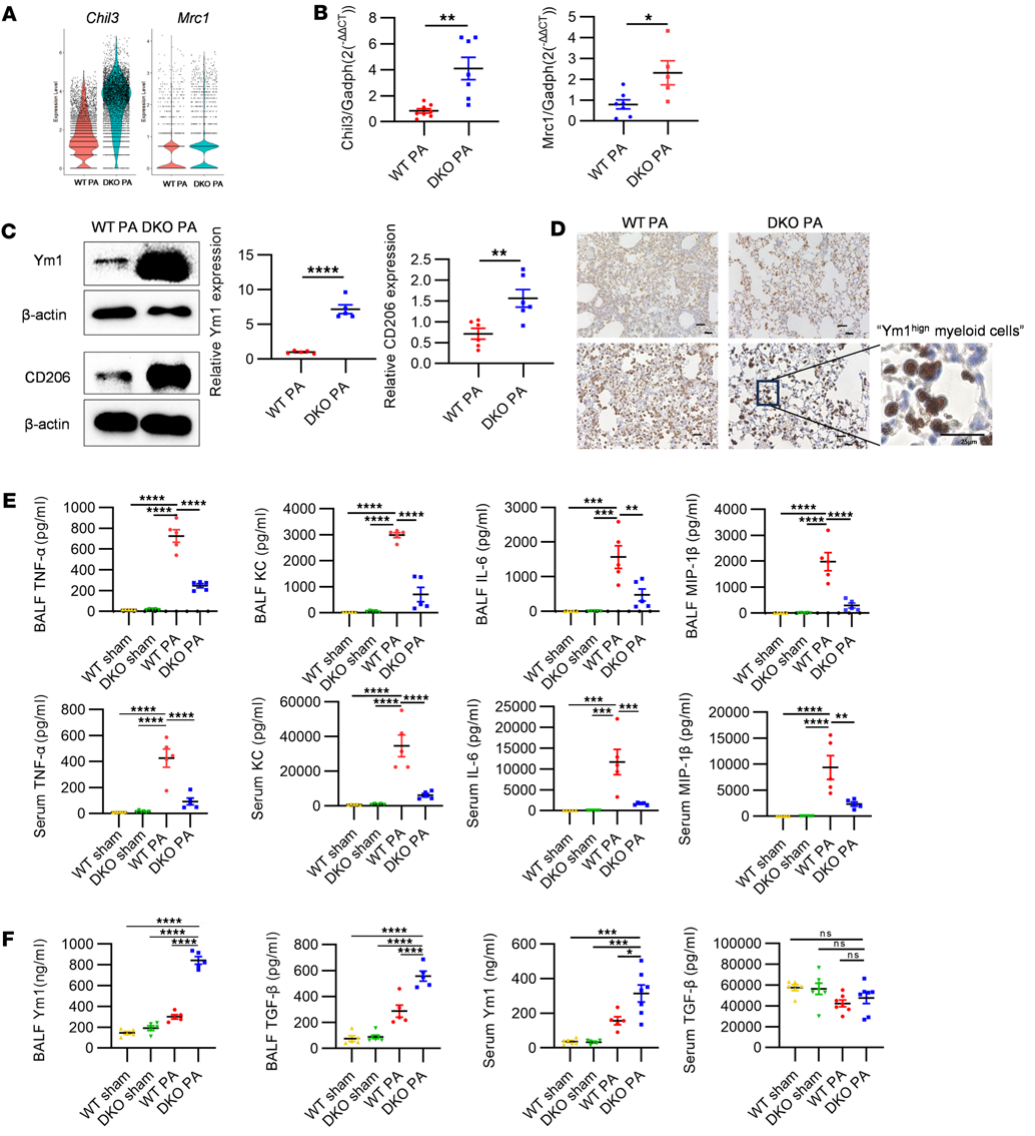
Resolution of inflammation prompted by *Chil3*^+^ myeloid cells through differentiation into M2 macrophages. (**A**) Violin plots depicting the expression levels of *Chil3* and *Mrc1* genes across myeloid cell clusters in the WT PA and DKO PA groups (*n* = 3 mice/group). (**B**) qPCR analysis of *Chil3* and *Mrc1* gene expression in BALF cell lysates from WT and DKO mice 24 hours after PA inoculation (*n* = 5–8 mice/group). (**C**) Western blot analysis for Ym1 and CD206 proteins in BALF cell lysates from WT and DKO mice 24 hours after PA inoculation (*n* = 5–6 mice/group). Relative protein expression levels are shown on the right panel. (**D**) IHC staining for Ym1 in lung tissues of WT and DKO mice within the PA-induced sepsis group. A zoomed-in view reveals Ym1^hi^ myeloid cell from DKO mice (*n* = 3 mice/group). Scale bars: 50 μm (upper panels); 25 μm (lower panels). (**E**) ELISA results showing concentrations of M1-related markers (TNF-α, KC, IL-6, and MIP-1β) in the BALF and serum of WT and DKO mice 24 hours after PA inoculation (*n* = 5 mice/group). (**F**) ELISA results showing concentrations of M2-related markers (Ym1 and TGF-β) in the BALF and serum of WT and DKO mice 24 hours after PA inoculation (*n* = 5–7/group). Results in **B**–**F** were representative of at least 3 independent experiments. Data for all bar charts were analyzed using unpaired Student’s *t* tests or 1-way ANOVA. Data are presented as means ± SEM. *P < 0.05; **P < 0.01; ***P < 0.001; ****P < 0.0001.

After PA inoculation, our scRNA-Seq analysis highlighted a notable increase in *Chil3* and *Mrc1* gene expression among myeloid cells in DKO mice relative to WT counterparts (Figure 5A). Subsequent validation via quantitative PCR (qPCR) (Figure 5B) and Western blot (Figure 5C) on BALF cell lysates from WT and DKO mice corroborated the scRNA-Seq findings, showing elevated levels of Ym1 and CD206. IHC staining further revealed a pronounced recruitment of Ym1^hi^ myeloid cells in lung tissues of DKO mice under PA-induced ALI conditions compared with WT mice, visually confirming the in situ manifestation of this M2 differentiation trend (Figure 5D).

ELISA assays for cytokines and chemokines associated with M1 and M2 phenotypes in BALF and serum were performed among all experimental groups. While proinflammatory markers (TNF-α, IL-6, KC, MIP-1β) were elevated in both WT PA and DKO PA mice, the levels were mitigated in DKO PA mice, illustrating a subdued inflammatory response (Figure 5E). Conversely, Ym1 and TGF-β as M2 markers (36), specifically Ym1, were higher in DKO mice for both BALF and serum, with TGF-β levels also increased in BALF (Figure 5F). This differentiation, driven by *Padi2* and *Padi4* deletion, appears pivotal in curtailing the excessive inflammation typically observed during PA-induced ALI.

### Regulation of the NLRP3/Ym1 pathway by DKO of Padi2 and Padi4 genes.

**Figure 6:**
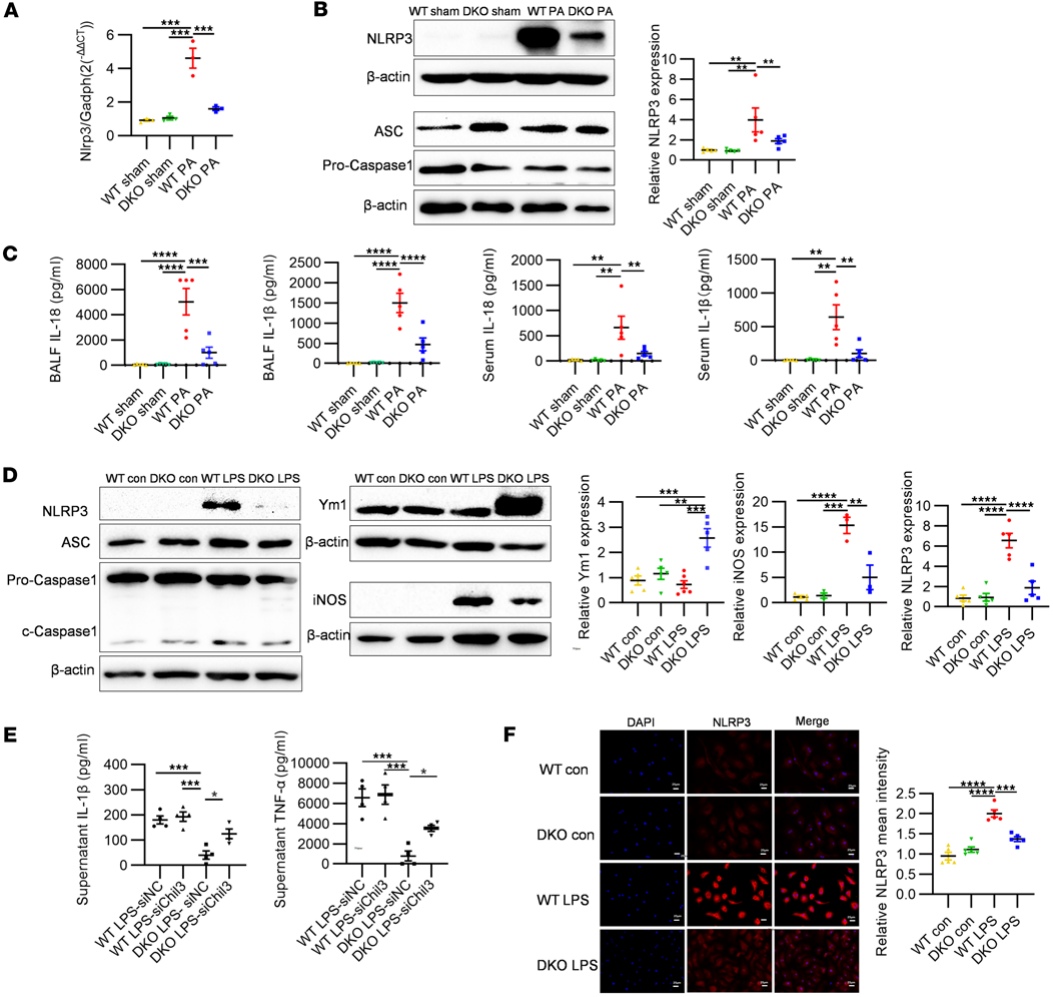
Regulation of the NLRP3/Ym1 pathway by DKO of *Padi2* and *Padi4* genes. (**A**) qPCR analysis of *Nlrp3* gene expression in BALF cell lysates from WT and DKO mice across sham and 24 hours after PA inoculation (*n* = 3 mice/group). (**B**) Western blot analysis demonstrating NLRP3, ASC, and Caspase-1 proteins expression levels in BALF cells from WT and DKO mice, both under sham conditions and 24 hours after PA inoculation (*n* = 5 mice/group). (**C**) ELISA results displaying concentrations of NLRP3 inflammasome–related cytokines (IL-1β and IL-18) in the BALF and serum of WT and DKO mice across sham and 24 hours after PA inoculation (*n* = 5 mice/group). (**D**) Western blot analysis showing the expression of Ym1, iNOS, NLRP3, ASC, and Caspase-1 proteins in bone marrow–derived macrophages (BMDMs) from WT and DKO mice, under both control conditions and after LPS treatment (250 ng/mL, 24 hours, *n* = 3–5/group). (**E**) ELISA results showing concentrations of IL-1β and TNF-α in cell culture supernatants of negative control siRNA (siNC) or *Chil3*-knockdown siRNA (siChil3) transfected WT BMDMs and DKO BMDMs after 24 hours of LPS treatment (*n* = 4/group). (**F**) Immunofluorescence analysis of NLRP3 expression in BMDMs from WT and DKO mice treated with LPS (250 ng/mL, 24 hours) compared with control (*n* = 5/group), with cells stained for NLRP3 (in red) and nuclei counterstained with DAPI (in blue). Results are representative of 3 independent experiments. Data for all bar charts were analyzed using 1-way ANOVA and are presented as means ± SEM. *P < 0.05; **P < 0.01; ***P < 0.001; ****P < 0.0001. Scale bars: 25 μm.

Our investigations into the DKO mice have revealed a substantial modulation of the NLRP3/Ym1 pathway during sepsis. qPCR analyses of BALF cell lysates demonstrates that there was a notable elevation in *Nlrp3* gene expression after PA infection, which was substantially reduced in DKO mice compared with WT counterparts, suggesting a regulatory effect of PADI2 and PADI4 on *Nlrp3* expression (Figure 6A). This trend was followed in protein levels, as confirmed by Western blot, indicating a consistent attenuation of the NLRP3 expression in DKO mice (Figure 6B). However, the expression levels of the NLRP3 inflammasome–related adaptor protein ASC (apoptosis-associated speck-like protein containing a caspase recruitment domain) and Caspase-1 showed no significant differences between WT and DKO mice (Figure 6B). Further examination of IL-1β and IL-18 cytokines, which are downstream of NLRP3 inflammasome pathway (37), through ELISA of BALF and serum samples, showed a similar pattern. Both cytokines were dramatically elevated after PA infection, yet their increase was significantly less pronounced in the DKO group (Figure 6C).

To elucidate the inverse relationship between NLRP3 and Ym1 during monocyte/macrophage differentiation and polarization, we utilized bone marrow–derived macrophages (BMDMs) from both WT and DKO groups, treated with PA-deprived LPS. Western blot analyses of these cells revealed that, while NLRP3 and inducible nitric oxide synthase (iNOS) were upregulated following LPS stimulation, their levels were markedly lower in the DKO group (Figure 6D), and the expression levels of ASC and Caspase-1 proteins still showed no significant differences between WT and DKO groups (Figure 6D). Immunofluorescence analysis corroborated these findings, displaying a subdued NLRP3 expression in DKO BMDMs after LPS treatment (Figure 6F). On the other hand, Ym1 expression significantly increased in DKO mice but not in WT controls under LPS stimulation (Figure 6D).

**Figure 7:**
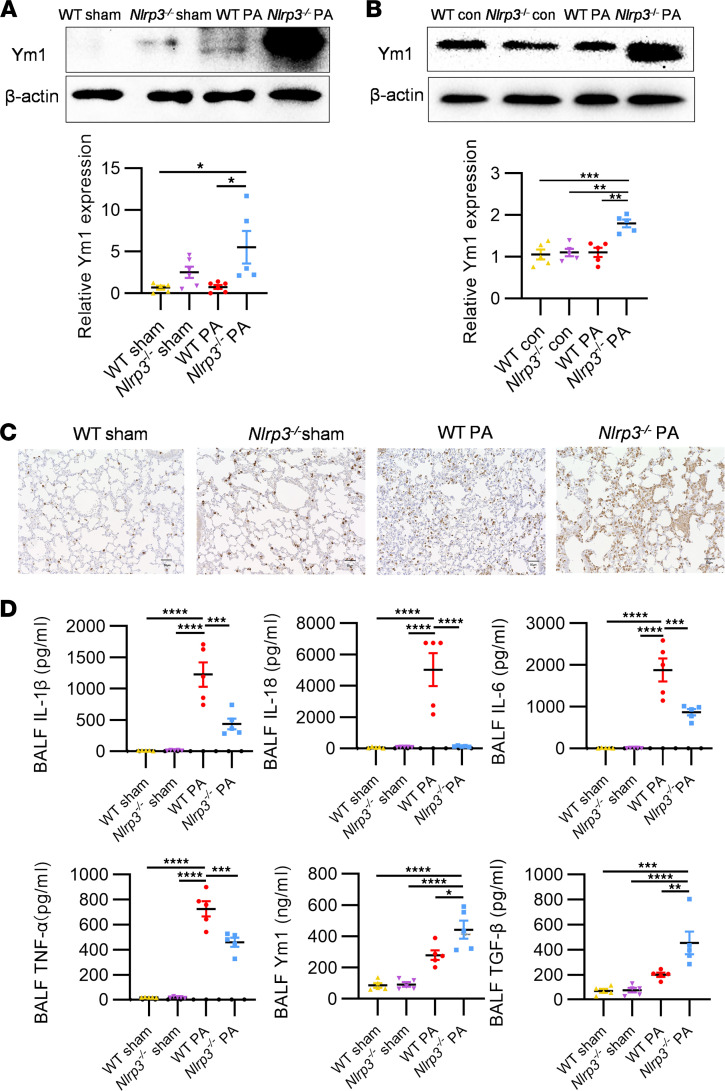
Validation of the NLRP3/Ym1 axis in *Nlrp3*-KO mice. (**A**) Western blot analysis comparing the expression of NLRP3 and Ym1 proteins in BALF cell lysates from WT and *Nlrp3^–/–^* mice, both under sham conditions and 24 hours after PA inoculation (*n* = 5–6 mice/group). (**B**) Western blot analysis comparing the expression of NLRP3 and Ym1 proteins in BMDMs from WT and *Nlrp3^–/–^* mice, following treatment with PA bacteria (MOI, 100; 1 hour) versus control (*n* = 5/group). (**C**) IHC staining depicting Ym1 expression and localization in lung tissue from WT and *Nlrp3^–/–^* mice (*n* = 3 mice/group). (**D**) ELISA results showing levels of NLRP3 inflammasome–related cytokines (IL-1β and IL-18), M1-related cytokines (TNF-α and IL-6), and M2-related cytokines (Ym1 and TGF-β) in BALF from WT and *Nlrp3^–/–^* mice, across sham conditions and 24 hours after PA inoculation (*n* = 5 mice/group). Results are representative of 3 independent experiments. Data for all bar charts were analyzed using 1-way ANOVA and are presented as means ± SEM. *P < 0.05; **P < 0.01; ***P < 0.001; ****P < 0.0001. Scale bars: 50 μm.

To elucidate the regulatory role of Ym1 in inflammation, we transfected BMDMs with siRNA to knock down *Chil3* expression at the RNA level, leading to a subsequent reduction in Ym1 protein expression (Supplemental Figure 4). IL-1β and TNF-α levels in the cell culture supernatant were then examined. The results demonstrated that BMDMs from DKO mice showed an inhibition of IL-1β and TNF-α cytokine release upon LPS treatment compared with WT groups. Notably, knockdown of *Chil3* in these cells restored the release of IL-1β and TNF-α cytokines in DKO mice (Figure 6E). Lastly, to explicitly delineate the NLRP3/Ym1 regulatory axis, we integrated *Nlrp3^–/–^* mice into our study, aiming to directly observe the effects of *Nlrp3* deletion on Ym1 expression and subsequent inflammatory response modulation. Western blot analysis of BALF cell lysates in *Nlrp3^–/–^* mice revealed a marked upsurge in Ym1 expression compared with both the sham control and WT groups subjected to the same PA challenge (Figure 7A). Further substantiation came from treating BMDMs from *Nlrp3^–/–^* and WT mice with PA bacteria. The *Nlrp3^–/–^* group exhibited elevated Ym1 levels after infection (Figure 7B). IHC staining of lung tissues from *Nlrp3^–/–^* mice revealed an increased recruitment of Ym1^hi^ myeloid cells after PA infection (Figure 7C). ELISA analyses of cytokines in BALF from WT and *Nlrp3*^–/–^ mice confirmed M1-related cytokines (IL-1β, IL-18, TNF-α, IL-6) were lower in *Nlrp3*^–/–^ mice, whereas M2-related cytokines (Ym1, TGF-β) were elevated, further illustrating the shift toward an antiinflammatory phenotype in the absence of NLRP3 (Figure 7D).

## Discussion

This study investigated the roles of PADI2 and PADI4 enzymes in regulating the immune response during sepsis, particularly focusing on their influence on the critical balance between proinflammatory and antiinflammatory pathways. AMs and the NLRP3 play key roles in determining the immune landscape following sepsis, with implications for lung injury (38–40). Our research aimed to elucidate how *Padi2* and *Padi4* deletions affect these mechanisms, revealing that such deletions enhance survival rates and mitigate lung injury in a PA pneumonia–induced sepsis mouse model (Figure 1, A and B).

Focusing on the lung, our scRNA-Seq analyses unveiled notable shifts in immune cell landscapes, particularly in AMs and myeloid cells in PA-induced ALI (Figure 2, A, B, and C), which pivotally orchestrate the pulmonary response to pathogens (41). However, there is still a lack of clear findings about how AMs react to pathogens, the change in their functions, and how they are replenished after phagocytosis and death in alveoli (42). We observed that, following PA infection, the deceased AMs appeared to be replenished by recruited myeloid cells (Figure 2, B and C), which demonstrated a lower phagocytosis capability compared with mature macrophages (42, 43). *Padi2* and *Padi4* deletion led to an increase in mature AMs after PA infection (Figure 2, D and E), potentially improving bacterial clearance and reducing sepsis-induced ALI. Our investigation highlighted the dynamic changes within AM populations after infection, revealing the emergence of a proinflammatory *Nlrp3*^hi^ AM subset (C12), potentially exacerbating lung injury (Figure 3, D and E). *Padi2* and *Padi4* deletion resulted in a decrease in the C12 *Nlrp3*^hi^ AMs population (Figure 3C), further reducing the production of proinflammatory mediators (Figure 3F). In vivo and in vitro experiments confirmed that deletion of *Padi2* and *Padi4* could inhibit NLRP3 expression in type 1 immune response (Figure 6, B and D), suggesting the role of PADI2 and PADI4 in regulating expression of NLRP3.

Macrophages, as key players in the immune response to sepsis, differentiate into M1-like macrophages, which promote inflammation, and M2-like macrophages, crucial for resolving inflammation (44, 45). It has been reported that silencing *PADI2* with *PADI2* siRNA shifted THP-1 macrophage polarization toward an M2-like phenotype (46). However, this was limited to use of the THP-1 cell line in vitro. Here, we firstly utilized DKO mice to investigate the role of PADIs in an in vivo sepsis-induced ALI model. Moreover, the precise location and origin of the M1 to M2 macrophage transition following sepsis — whether it occurs within the alveoli from resident macrophages, from recruited myeloid cells, or a combination of both — remain to be determined (47). In our study, an increase in resident *Nlrp3*^hi^ macrophages indicated a shift toward M1 polarization after sepsis. Simultaneously, a marked increase in recruited myeloid cells was observed in the alveoli, exhibiting M1/M2 polarization potential (Figure 2B and Figure 4D). After deletion of *Padi2* and *Padi4*, we identified a predominant presence of *Chil3*^hi^ myeloid cells (C2, C13), indicative of a shift toward M2 macrophage development (Figure 4, D–G). Intriguingly, the deletion of *Padi2* and *Padi4* modulated this myeloid-macrophage differentiation and polarization, consequently altering the inflammatory milieu within the lung after sepsis. This was further corroborated by the altered profiles of inflammatory and antiinflammatory mediators (Figure 5, E and F), indicating a systemic shift toward a resolution of inflammation in DKO mice.

Research indicates that NLRP3 expression is upregulated in myeloid cells upon exposure to proinflammatory stimuli, initiating an inflammatory state (48, 49). The NLRP3 inflammasome, composed of NLRP3 protein, is known to drive M1 polarization (50). Notably, the activation and efficacy of the NLRP3 inflammasome largely depend on NLRP3 expression itself (51, 52), despite a proinflammatory environment not necessarily triggering other inflammasome components including ASC and Caspase-1 proteins (Figure 6, B and D). This suggests that NLRP3 protein might independently promote M1 polarization without activation of NLRP3 inflammasome. Our findings reveal that deletion of *Padi2* and *Padi4* could induce novel *Nlrp3*^lo^*Chil3*^hi^ myeloid cell populations (C2, C13), indicative a shift from M1 to M2 phenotype (Figure 5, E and F). The interaction between the *Nlrp3* and *Chil3* genes shows antagonistic expression patterns in AMs and myeloid cells (Figure 4, H and I). Evidence suggests that *Nlrp3* deletion enhances Ym1 expression and M2 macrophage activation following helminth infection, which is typically associated with type 2 inflammatory responses (53). Here we provide evidence that NLRP3 directly affects Ym1 expression in myeloid differentiation and polarization after PA infection. Our findings indicate that *Padi2* and *Padi4* deletions could suppress NLRP3 and iNOS-related M1 polarization while promoting Ym1-related M2 polarization expression (Figure 6D).

Moreover, the use of siRNA to knock down *Chil3* solidifies the role of Ym1 as a key downstream regulator of inflammatory cytokines, as the reduction in *Chil3* expression directly affected the levels of IL-1β and TNF-α (Figure 6E). Further analysis using *Nlrp3^–/–^* mice confirmed that *Nlrp3* deletion increased Ym1 expression both in vivo and in vitro after PA infection (Figure 7, A–C), indicating an immune shift from proinflammatory to antiinflammatory (Figure 7D), thereby facilitating tissue repair and remodeling. Overall, our results reveal a mechanism by which the PADIs/NLRP3/Ym1 pathway influences macrophage behavior in sepsis, suggesting that targeting this pathway could alleviate the inflammatory response in PA-induced ALI and improve survival.

However, our study acknowledges limitations in fully understanding how PADI2 and PADI4 modulate NLRP3 and macrophage polarization. Future research should delve into the effects of citrullination, a posttranslational modification influenced by these enzymes, on NLRP3 expression and functionality (54, 55). Investigating other inflammatory models could help validate the PADIs/NLRP3/Ym1 axis’s role beyond the context of PA-induced ALI, potentially uncovering broader therapeutic targets for inflammatory diseases.

In conclusion, our results demonstrate that *Padi2* and *Padi4* deletion can mitigate lung injury and improved survival of septic mice. This protective effect was achieved by suppressing NLRP3 expression, which in turn encouraged the differentiation of Ym1^+^ myeloid cells into M2 macrophages, promoting a shift from an excessively activated inflammatory state toward the resolution of inflammation. This mechanism underscores the therapeutic potential of targeting the PADIs/NLRP3/Ym1 axis in sepsis treatment, offering a promising strategy for treating patient with sepsis and/or other related inflammatory disorders.

## Methods

### Sex as a biological variable.

Our study examined male and female animals, and similar findings are reported for both sexes. All mice used for in vivo and in vitro experiments were matched for age and sex (8–12 weeks old).

### Animals.

The DKO mice were generated using CRISPR/Cas9 technology by Michigan Diabetes Research Center (MDRC) Molecular Genetics Core (MGC). These DKO mice were generated via the insertion of a premature stop codon or frameshift mutation by insertion or deletion (indel) near the start codon of each exon (*Padi2* and *Padi4*), thereby halting translation following residue 10 (*Padi4*) and residue 22 (*Padi2*). Subsequently, these generated DKO mice were mated with WT mice of a C57BL/6J background to establish germline transmission. Genotyping results and validation of *Padi2* and *Padi4* expression confirmed the KO of both *Padi2* and *Padi4* genes.

Male and female C57BL/6J WT mice were obtained from The Jackson Laboratory. WT animals were acclimatized in our pathogen-free animal facility for 3 days prior to any experimental procedures. *Nlrp3*-KO (*Nlrp3*^–/–^) mice were provided by Gabriel Nunez (University of Michigan, Ann Arbor, Michigan, USA).

### Pulmonary infection model.

Mice were exposed to intranasal administration of PA (19660; ATCC) solution to induce PA pneumonia–induced sepsis. Briefly, a PA solution was prepared at a concentration of 8.25 × 10^7^ CFU/mL in PBS (Thermo Fisher Scientific). The mice were anesthetized with ketamine (Dechra Veterinary Products) and xylazine (Akorn), and they were then held vertically. Subsequently, 15 μL of the PA solution was instilled into each nostril, totaling 30 μL to achieve a final bacterial load of 2.5 × 10^6^ CFU. Mice inoculated with sterile PBS served as sham controls. In nonsurvival studies, mice were euthanized by CO_2_ 24 hours after inoculation. In survival studies, WT and DKO mice were monitored for 10 days, after which they were euthanized with CO_2_ either at the designated endpoint of observation or when they were found moribund.

### ALI assessment.

The lungs of mice were harvested either 24 hours after sham treatment or following PA infection. Tissues were fixed in 4% neutral-buffered formaldehyde and subsequently embedded in paraffin. Lung tissue sections were stained with H&E and graded by a board-certified pathologist who was blinded to the experimental conditions.

### Bacterial load determination.

Mice were euthanized 24 hours after PA infection, and bronchoalveolar lavage was performed on both WT and DKO mice by flushing with PBS to yield 5 mL of aspirate fluid. Blood samples were collected via cardiac puncture. Samples of blood and BALF were serially diluted by 10-fold in PBS. Subsequently, 10 μL of each blood and BALF sample were plated onto nutrient agar (213000, BD Biosciences) plates and incubated at 37°C for 20 hours. The number of bacterial colonies was then counted from the plates. Results were expressed as CFU per mL of blood and BALF.

### Sample preparation and scRNA-Seq.

BALF samples were collected from both WT and DKO mice after PA infection and sham control. BALF samples from 3 mice per group were pooled to generate samples (*n* =3 mice/sample) for the isolation of BALF cells. The BALF samples were centrifuged for 5 minutes at 400*g* and 4°C. The cell pellet was then resuspended in 500 μL of RBC lysis buffer (00-4333-57, eBioscience) and incubated for 5 minutes at room temperature. Following this, 500 μL of cold PBS was added to dilute the RBC lysis buffer, and the mixture was centrifuged for 5 minutes at 400*g* and 4°C. The resulting single cells were resuspended in PBS containing 1% weight/volume FBS (Thermo Fisher Scientific), and cell viability was determined using automated cell counters (Invitrogen). The single-cell suspension was thoroughly mixed and loaded onto a 10X Chromium system to capture no more than 10,000 single cells using the Chromium Next GEM Single Cell 3′ GEM, Library & Gel Bead Kit (10X Genomics). The cells were partitioned into Gel Beads in the Chromium instrument. DNA amplification and library construction were performed with cell lysis and barcoded reverse transcription of RNA. The resulting libraries were sequenced using an Illumina HiSeq 4000 next-generation sequencing platform. Data quality analysis and mapping to ensemble gene symbols were conducted using CellRanger (10X Genomics).

### scRNA-Seq data processing.

The CellRanger output data were imported into the Seurat R package (version 5.0.1) for unsupervised clustering analysis. Prior to clustering, filtering procedures were implemented to eliminate multiplets and damaged cells, while sources of variation deemed uninformative were regressed out. Identification of variable genes was achieved through iterative selection based on the dispersion versus average expression profile of each gene. Normalization and scaling of gene expression values within individual cells were conducted using the SCTransform algorithm. Dimensionality reduction and visualization of the data were performed using principal components analysis and UMAP, incorporating the top 30 principal components. Parameters for UMAP were set to min.dist = 0.3 and n.neighbor = 30. Subsequently, cells were clustered using an unsupervised clustering approach with default parameters for the Seurat package (resolutio*n* = 0.6). Cluster-specific marker genes were identified utilizing the FindAllMarkers function in Seurat, with criteria set at *P* < 0.01 and log (fold change) > 0.25 within the target cluster. Visualization of gene expression patterns across cell clusters was accomplished using UMAP plots and heatmaps generated with functions available in the Seurat package. Differentially expressed genes (DEGs) were determined using the FindAllMarkers function with default parameters, specifying log (fold change) > 0.26, *P* < 0.01, and min.pct > 0.1. For gene enrichment analysis, DEGs across different cell types were utilized to conduct cluster-specific pathway enrichment analysis. Single-cell trajectory analysis was performed on macrophage, DC, and myeloid cell populations using Monocle3 (version 1.3.1). UMAP dimensional reduction was applied to visualize the cells, and pseudotemporal trajectories were learned using the learnGraph and orderCells functions to elucidate cell connections. Gene expression profiles of interest across pseudotime were visualized using the plot_genes_in_pseudotime function to assess cell trajectory dynamics.

### Flow cytometry.

The abundance and diversity of BALF cells were assessed using flow cytometry. Following RBC lysis, BALF cells were pelleted by centrifugation at 400*g* for 5 minutes and resuspended in FACS buffer composed of PBS supplemented with 1% FBS. Cells were then incubated with fluorophore-conjugated antibodies, including FITC-conjugated anti-F4/80 antibody (123107; BioLegend), Pacific blue–conjugated anti-CD11c antibody (117321; BioLegend), PE-conjugated anti-CD11b antibody (101217; BioLegend), and APC-conjugated anti–Gr-1 antibody (108411; BioLegend). Resident macrophages were identified by their FITC and PB double-positive signals, while recruited myeloid cells were identified by their PE and APC double-positive signals during flow cytometry analysis. Flow cytometry data were processed and analyzed using FlowJo Software.

### Measurement of cytokines and chemokines.

The levels of TNF-α, IL-6, MIP-1β, TGF-β, IL-10, Ym1, KC, IL-1β, and IL-18 in BALF, serum, and cell culture supernatants were measured by the core of UMICH Immune Monitoring Shared Resource using the core-developed sandwich ELISA.

### BMDM isolation and transfection.

BMDMs were isolated for in vitro experimentation. Tibiae and femurs were obtained from both WT and DKO mice. Bone marrow cells were collected and seeded in 75 mm^2^ petri dishes containing IMDM (Thermo Fisher Scientific) supplemented with 20% FBS, 1% penicillin/streptomycin (Lonza Inc), and 30% L929 cell (CCL-1; ATCC) supernatant. The L929 cell supernatant was generated by incubating L929 cell fibroblasts in IMDM with 10% FBS for 6 days to produce macrophage CSF (M-CSF). After 7 days, BMDMs were harvested and diluted in Opti-MEM (Thermo Fisher Scientific) to the desired concentrations.

For experiments involving LPS treatment, BMDMs were exposed to 250 ng/mL PA-deprived LPS (L9143; Sigma-Aldrich) in Opti-MEM for 24 hours, while control BMDMs were treated with Opti-MEM alone for the same duration. For experiments involving PA transfection, BMDMs were treated with Opti-MEM containing PA bacteria at a MOI of 100. Control BMDMs were treated with Opti-MEM alone for 1 hour.

### Real-time PCR.

Total RNA was extracted from cells by using RNeasy Mini kit (74106; Qiagen Science). qPCR was performed by using QuantiTech SYBR RT-PCR kit (204243; Qiagen Sciences). Transcript levels detected were normalized against that of *Gadph*. The primers are shown in Supplemental Table 1.

### Western blotting.

For Western blotting, cells were washed 2 times with ice-cold PBS and lysed with RIPA buffer (89900; Thermo Fisher Scientific) plus halt protease inhibitor cocktail (87787; Thermo Fisher Scientific) for 30 minutes on ice. After addition of 4× Laemmli sample buffer (Bio-Rad), samples were separated by 10% SDS-PAGE electrophoresis and transferred to nitrocellulose membrane (Bio-Rad), and proteins of interest were incubated overnight with diluted primary antibodies (CD206, 24595, Cell Signaling Technology; NLRP3, 15101, Cell Signaling Technology; iNOS, PA5-17106, Invitrogen; Ym1, PA5-81356, Invitrogen; β-actin, 4970, Cell Signaling Technology) and then incubated for 1 hour with a secondary antibody (31460, Invitrogen). The membranes were visualized using enhanced chemiluminescence (1705061; Bio-Rad) within the luminescent image analyzer (Thermo Fisher Scientific).

### IHC analysis.

Formalin-fixed, paraffin-embedded lung tissue sections were stained with the Ym1 antibody (Ab230610, Abcam) by the In-Vivo Animal Core at the University of Michigan. Digital images of the stained tissue sections were acquired using a KEYENCE BZ-X800 microscope.

### ICC analysis.

BMDMs were allowed to adhere to poly-l-lysine–coated glass coverslips for 15 minutes at 37°C. Subsequently, coverslips were blocked with a solution containing 5% BSA (Thermo Fisher Scientific) and 0.05% Triton X-100 (Thermo Fisher Scientific) in PBS for 1 hour. Immunostaining was conducted using an anti-NLRP3 antibody (768319, Invitrogen) at a dilution of 1:100 overnight, followed by incubation with a second antibody (ab150064, Abcam). Nuclear staining was achieved using DAPI (62248, Thermo Fisher Scientific). Microscopic imaging was performed using a KEYENCE BZ-X800 microscope.

### siRNA gene knockdown.

Knockdown experiments were performed using predesigned and validated siRNA for *Chil3* or a negative control siRNA (siNC, 4390843, Invitrogen). The sequences of primers are shown in Supplemental Table 1. BMDMs were seeded per well in 6-well plates 24 hours before transfection. After overnight incubation, BMDMs were transfected at 100 nM final concentration with siNC or siChil3 following Lipofectamine RNAi Max (Invitrogen) protocol. The cells were allowed to internalize the siRNA for 48 hours in Opti-MEM, after which the media were replaced with 250 ng/mL PA-deprived LPS media for 24 hours for determination of cytokines (IL-6 and TNF-α) by ELISA. Knockdown efficacy was analyzed by means of qPCR and Western blotting.

### Statistics.

All results are expressed as the means ± SEM for the data with GraphPad Prism. Statistical analysis was performed using 2-tailed unpaired *t* test or 1-way ANOVA. Survival data were analyzed by Kaplan-Meier analysis and log-rank test. A P value less than 0.05 was considered significant.

### Study approval.

All animal experiments were performed with approval from the University of Michigan (PRO00010569).

### Data availability.

Values for all data points for each graph are included in the Supporting Data Values file. All scRNA-Seq data were deposited in the NCBI’s Gene Expression Omnibus database (GEO GSE274823).

## Author contributions

Conceptualization was contributed by XY and YL; methodology was contributed by XY, YS, TD, WO, LS, KEL, and YL; formal analysis was contributed by XY, YL, and YS; investigation was contributed by XY, TD, WO, LS, CQ, and YS; writing of the original draft was contributed by XY, YS, and YL; review and editing were contributed by XY, YS, YL, TD, TT, AT, KK, and JM; visualization was contributed by XY, YS, TD, MZ, HBA, and YL; supervision was contributed by YL; project administration was contributed by XY and YL; and funding acquisition was contributed by YL.

## Supplementary Material

Supplemental data

Unedited blot and gel images

Supporting data values

## Version 1 10/15/2024

In-Press Preview

## Version 2 11/22/2024

Electronic publication
