# Supplementary material for: Loss of PADI2 and PADI4 ameliorates sepsis-induced acute lung injury by suppressing NLRP3+ macrophages
Source: JCI Insight. 2024 Nov 22;9(22):e181686. doi: 10.1172/jci.insight.181686 (PMC11601939; doi:10.1172/jci.insight.181686)
Supplement: Supplemental data [file jciinsight-9-181686-s210.pdf]

Supplemental information

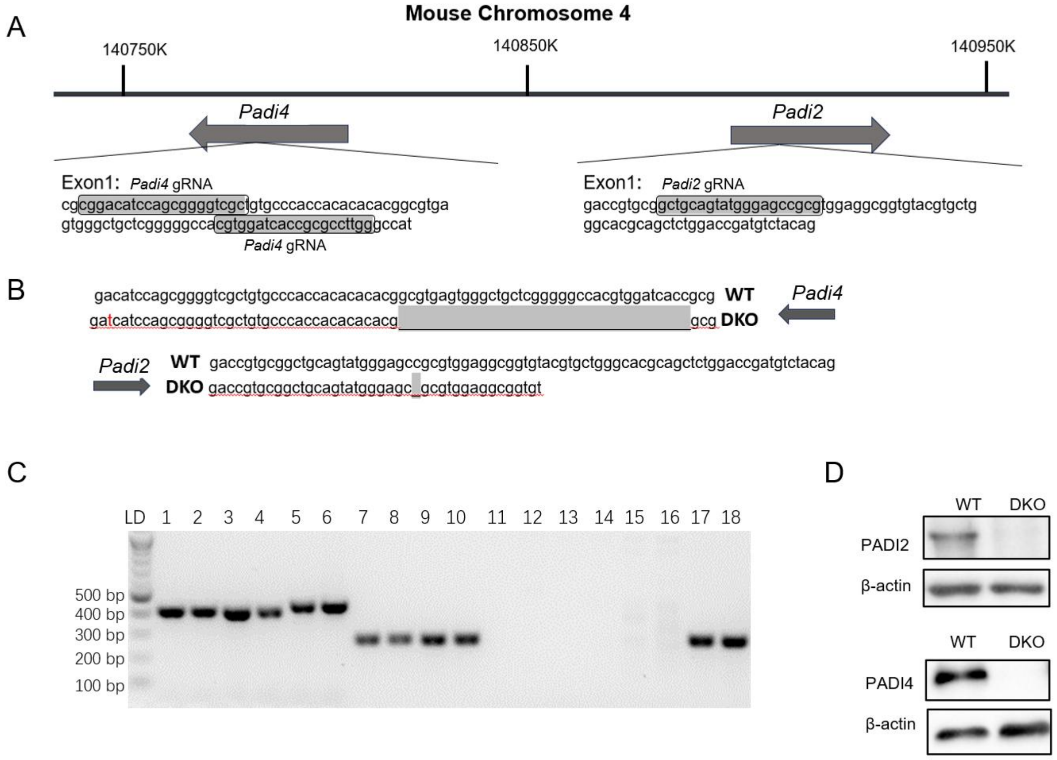

Supplemental Figure S1 Generation of *Padi2$^{-/-}$Padi4$^{-/-}$* mice

(A) Design and sequence of the guide RNA (gRNA) targeting Exon1 of the *Padi4* gene and Exon1 of the *Padi2* gene.

(B) Sanger sequencing confirmed a 36-nucleotide deletion and a 1-nucleotide insertion in the DKO *Padi4* Exon1. Sanger sequencing confirmed a 1-nucleotide deletion in the DKO *Padi2* Exon1.

(C) Representative picture shows result of genotyping. LD: DNA ladder; Lane 1-4: DKO mice for *Padi4*-KO primer; Lane 5-6: WT mice for *Padi4*-WT primer; Lane 7-10: DKO mice for *Padi2*-KO primer; Lane 11-12: WT mice for *Padi2*-KO primer; Lane 13-16: DKO mice for *Padi2*-WT primer; Lane 17-18: WT mice for *Padi2*-WT primer. Result analysis: (1) For *Padi2* KO: if it is a WT homozygote, then we will see a band only in the lane which was loaded with *Padi2*-WT primer-induced products; and if it is a *Padi2*-KO homo, we will see a band only in the lane which was loaded with *Padi2*-KO primer-induced products. (2) For *Padi4* KO: if it is a WT homo, we will find the band at the size around 450 bp (which is higher than that of *Padi4* KO DNA); if it is a *Padi4* KO homo, we will see one band at the size around 410 bp.

(D) Western Blot result shows PADI2 and PADI4 expression in bone marrow derived cells from a WT mouse and a DKO mouse.

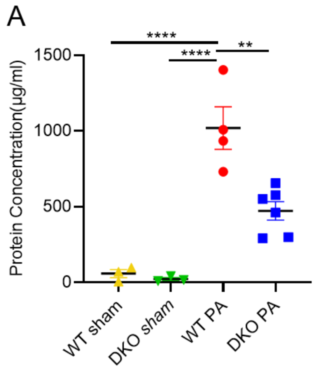

Supplemental Figure S2. Total protein concentrations of BALF from WT and DKO mice

(A) Total protein concentrations of BALF from WT and DKO mice 24 hours after PA infection and sham condition (n = 4-6). Data were analyzed using one-way analysis of variance (ANOVA). Data are presented as means ± SEM. **P < 0.01, ****P < 0.0001.

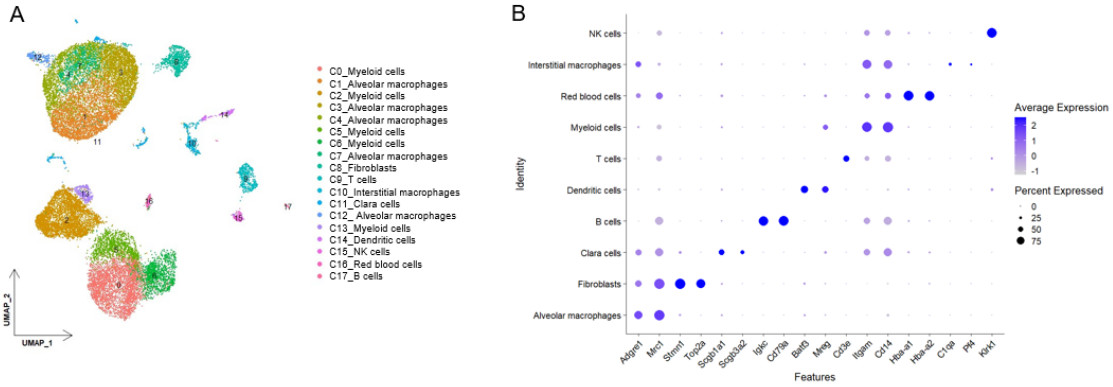

Supplemental Figure S3. Cell clusters of WT and DKO mice in alveoli

(A) UMAP plots of 22917 BALF cells allocated into 18 clusters from 6 WT mice and 6 DKO mice at PA-induced sepsis and control. BALF cells from 3 mice were mixed as one sample.

(B) Ten different clusters and their specific marker gene expression levels, where brightness indicates log-normalized average expression, and circle size indicates percent expressed.

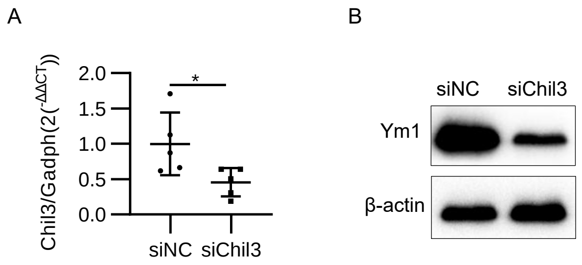

Supplemental Figure S4. Validation of *Chil3* knockdown efficacy with siRNA

(A) qRT-PCR result shows *Chil3* knockdown BMDMs using siChil3 have decreased expression of *Chil3* compared with negative control (siNC) transfected cells.
(B) Western Blot result shows *Chil3* knockdown BMDMs using siChil3 have decreased expression of Ym1 protein compared with siNC transfected cells.

Tabel S1

**primers**	**Sources**	**Identifier**
*Padi2*_WT-F-Mouse, 5'-GGCTGCAGTATGGGAGCC-3'(sense) and 5'-CTCAGGATTGCATGGAAGTGG-3'(antisense)	Intergrated DNA Technologies	NA
*Padi2*_KO, 5'-GGCTGCAGTATGGGAGCG-3'(sense) and 5'-CTCAGGATTGCATGGAAGTGG-3'(antisense)	Intergrated DNA Technologies	NA
*Padi4*_KO, 5'-CACAGAGAACTAATTGGCACGATAGGC-3'(sense) and 5'-CTGTTGCAGGCTTCACTGTGGAC -3'(antisense)	Intergrated DNA Technologies	NA
Nlrp3, 5'-ATTACCCGCCCGAGAAAGG-3'(sense) and 5'-TCGCAGCAAAGATCCACACAG -3'(antisense)	Intergrated DNA Technologies	NA
Chil3, 5'-GCATTCCAGCAAAGGCATAG-3'(sense) and 5'-GCTAAGGACAGGCCAATAGAA-3'(antisense)	Intergrated DNA Technologies	NA
Mrc1, 5'-CTCTGTTCAGCTATTGGACGC -3'(sense) and 5'-CGGAATTTCTGGGATTCAGCTTC -3'(antisense)	Intergrated DNA Technologies	NA
Gadph, 5'-TGCACCACCAACTGCTTAGC -3'(sense) and 5'-GGCATGGACTGTGGTCATGAG -3'(antisense)	Intergrated DNA Technologies	NA
**siRNA**	**Sources**	**Identifier**
siChil3, 5'-CAGGAAUCAUUGACGUAAUTT-3'(sense) and 5'-AUUACGUCAAUGAUUCCUGCT-3'(antisense)	Invitrogen	4390771
