# Supplementary material for: Loss of PADI2 and PADI4 ameliorates sepsis-induced acute lung injury by suppressing NLRP3+ macrophages
Source: JCI Insight. 2024 Nov 22;9(22):e181686. doi: 10.1172/jci.insight.181686 (PMC11601939; doi:10.1172/jci.insight.181686)

Full unedited blots of Figure 5C (For Ym1 protein)

Replicate 1

Ym1

loading control: β-actin

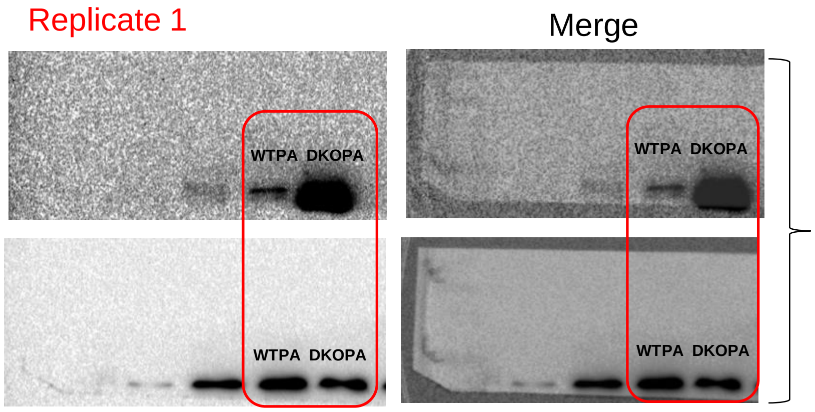

the same lanes of the same gel

Replicate 2

Ym1

loading control: β-actin

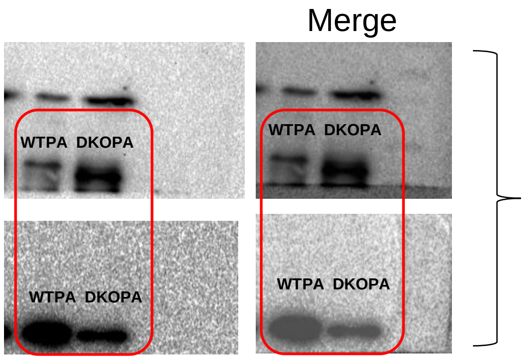

the same lanes of the same gel

Replicate 3

Ym1

loading control: β-actin

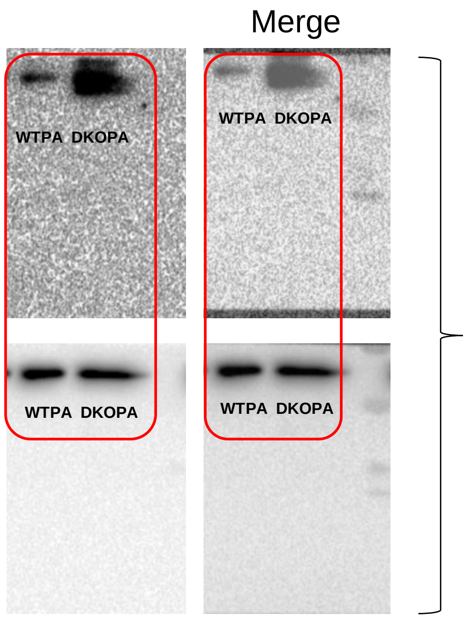

the same lanes of the same gel

Full unedited blots of Figure 5C (For CD206 protein)

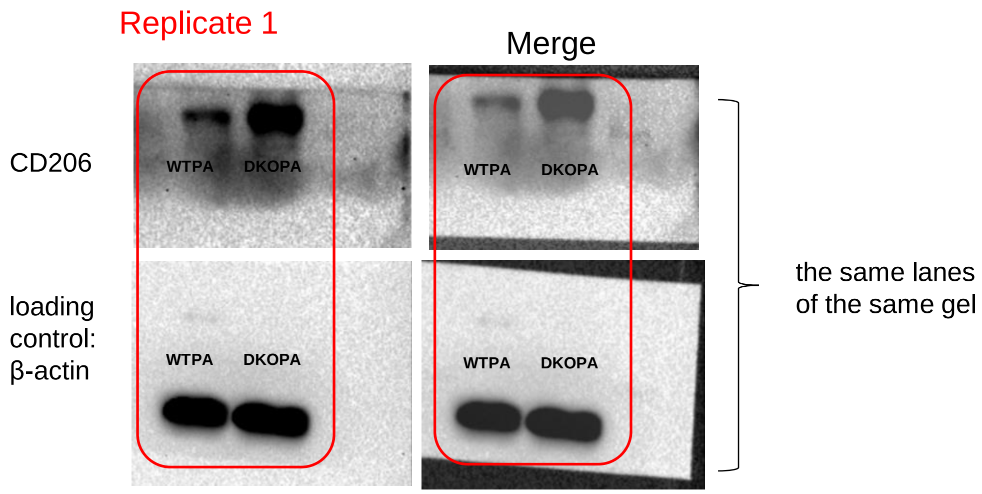

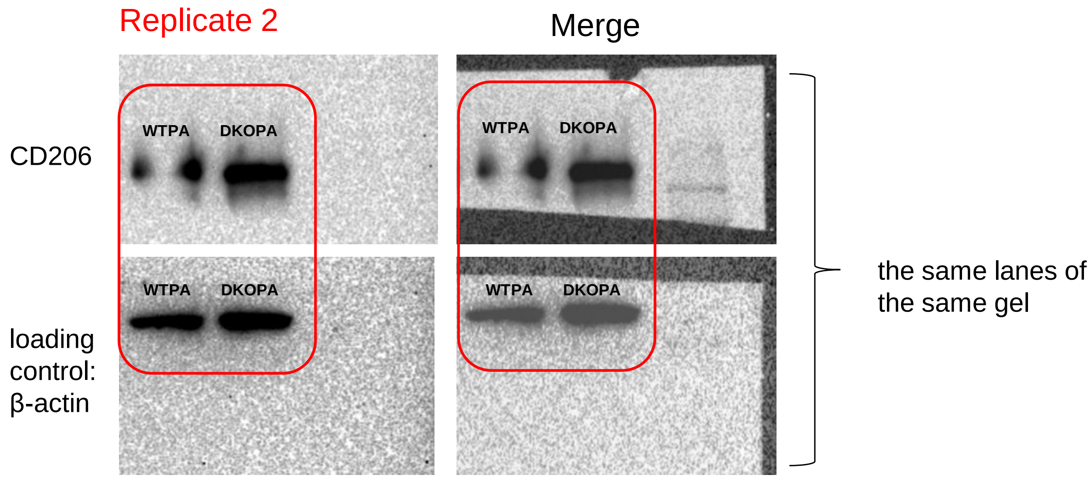

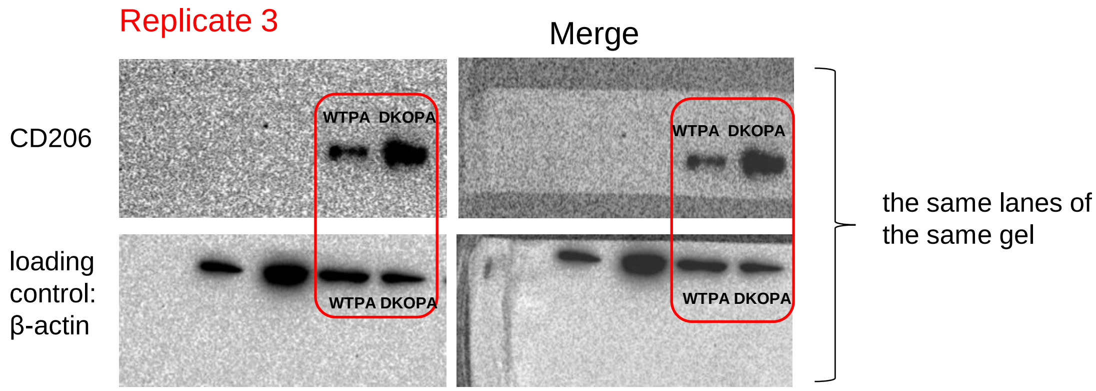

Full unedited blots of Figure 6B (For NLRP3 protein)

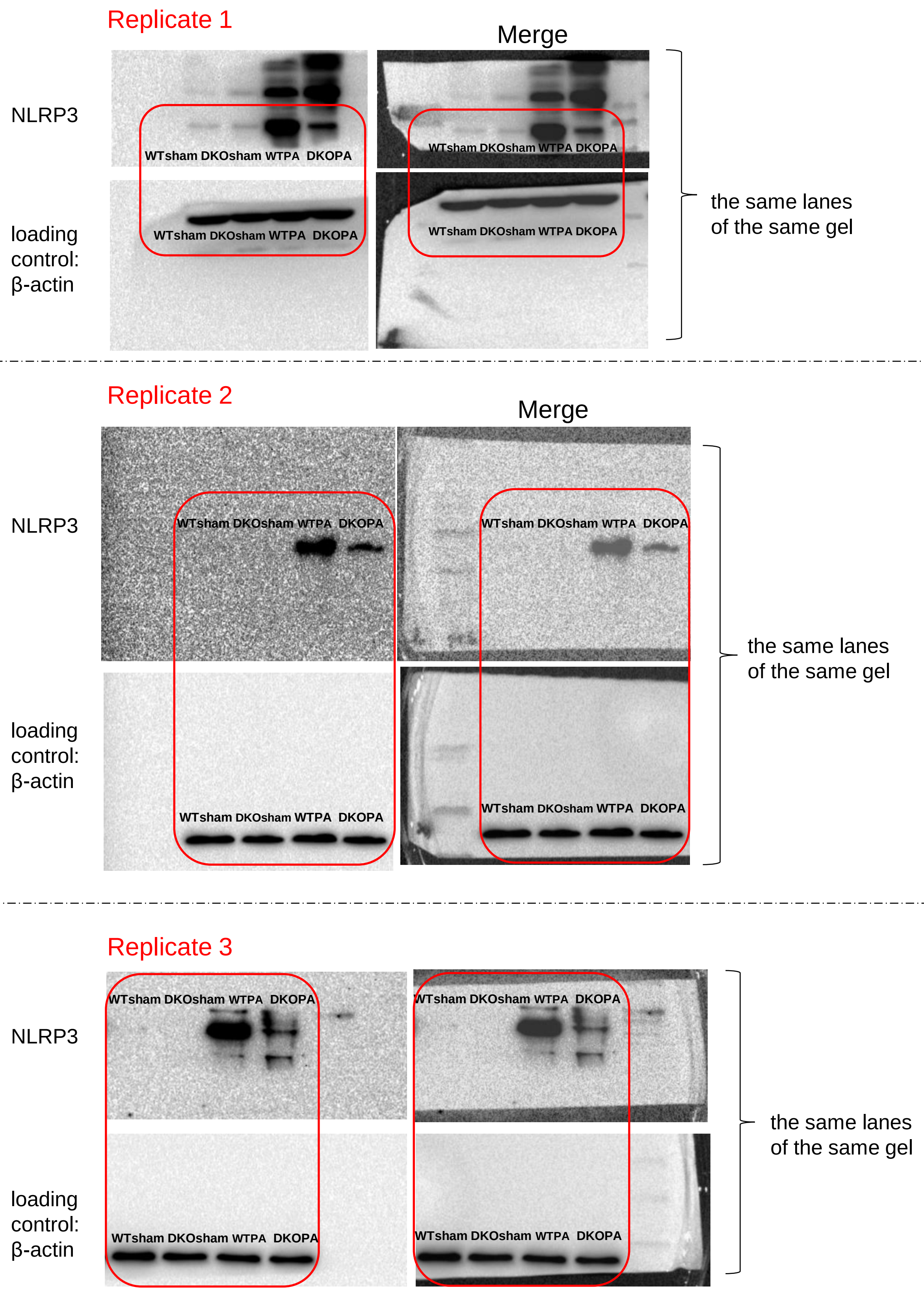

Full unedited blots of Figure 6B (For ASC & Caspase1 proteins)

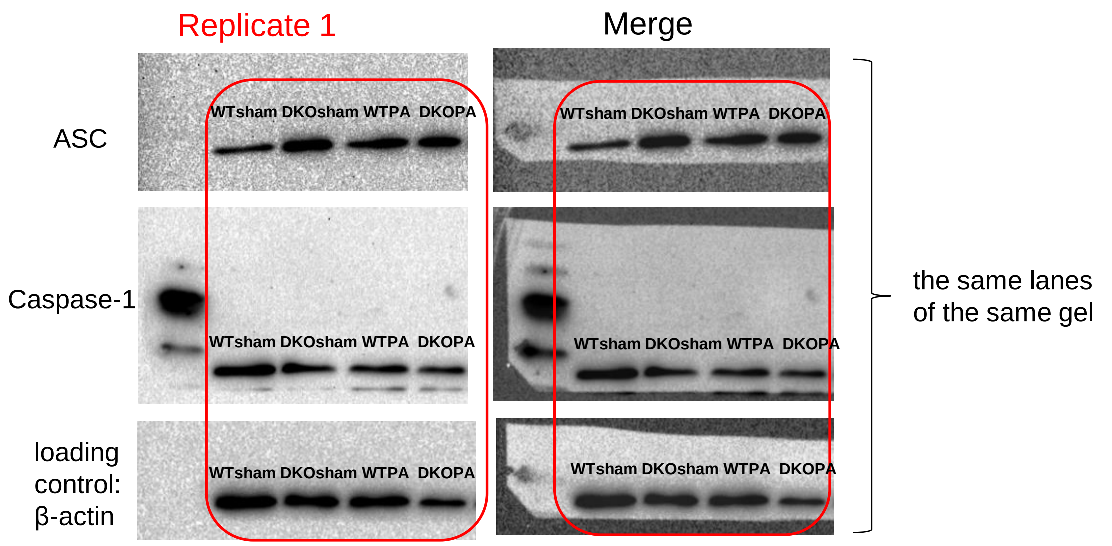

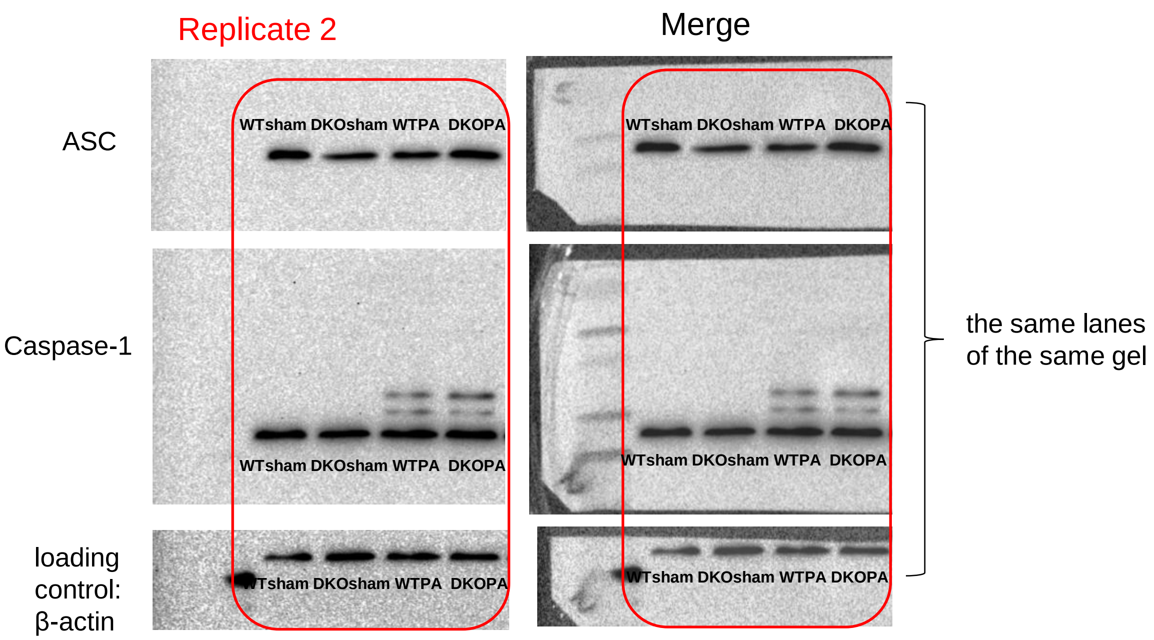

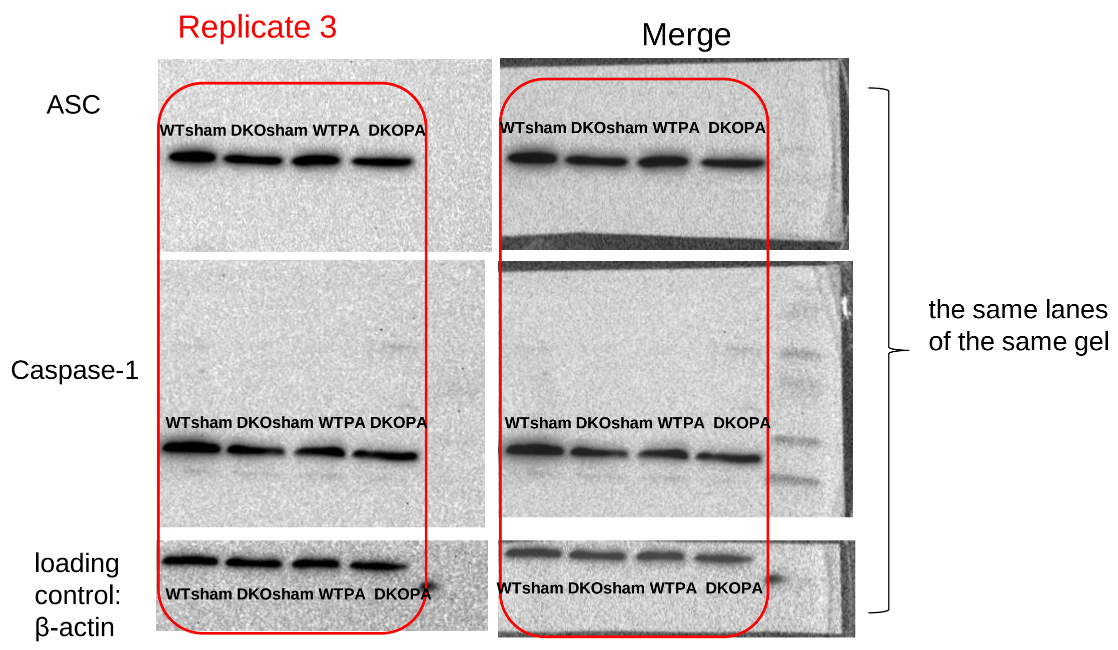

Full unedited blots of Figure 6D (For NLRP3 protein)

Replicate 1

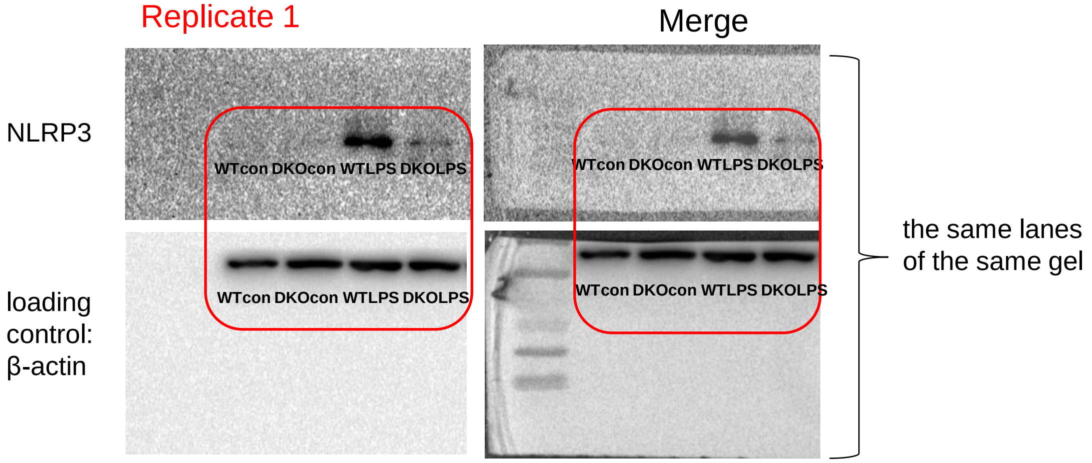

Replicate 2

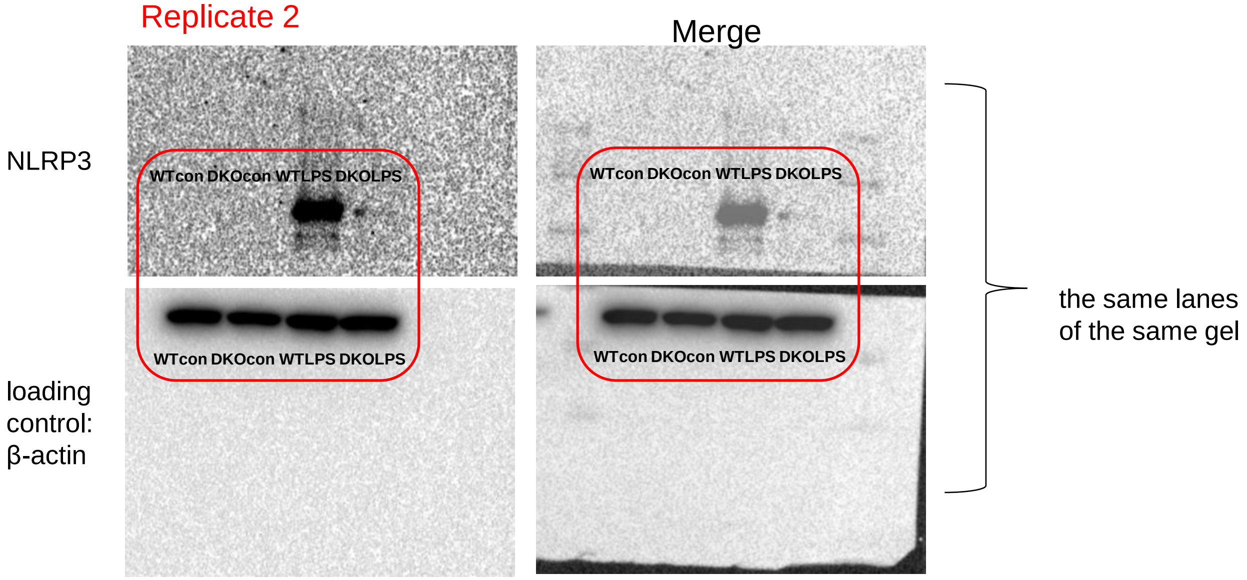

Replicate 3

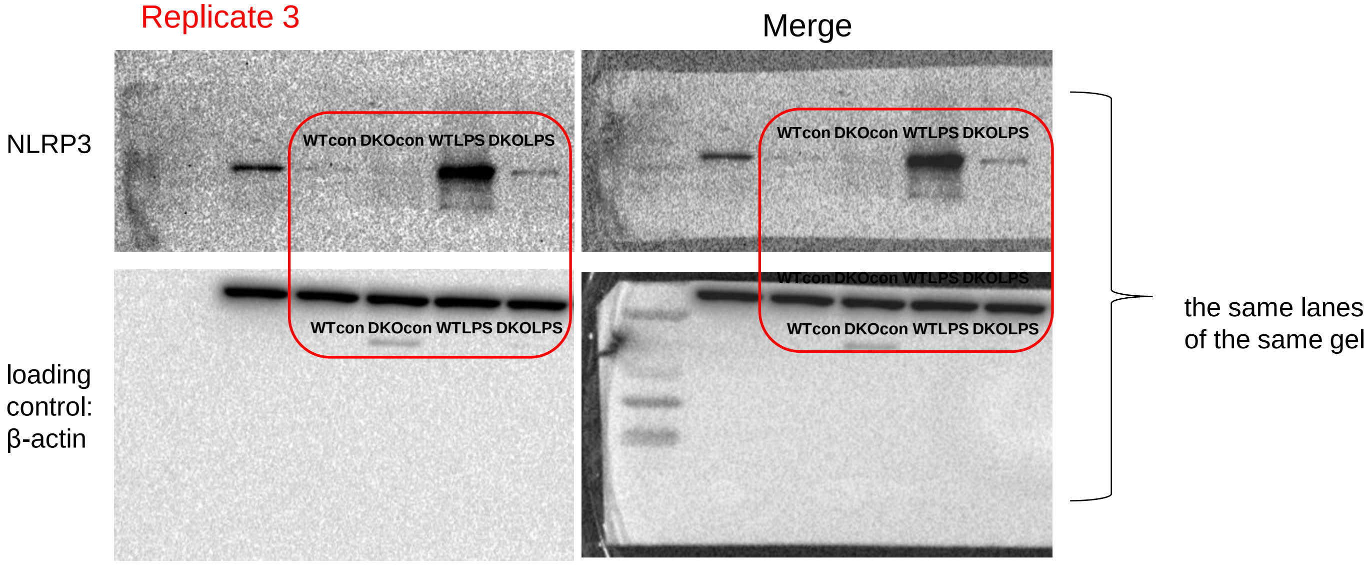

Full unedited blots of Figure 6D (For ASC & Caspase1 proteins)

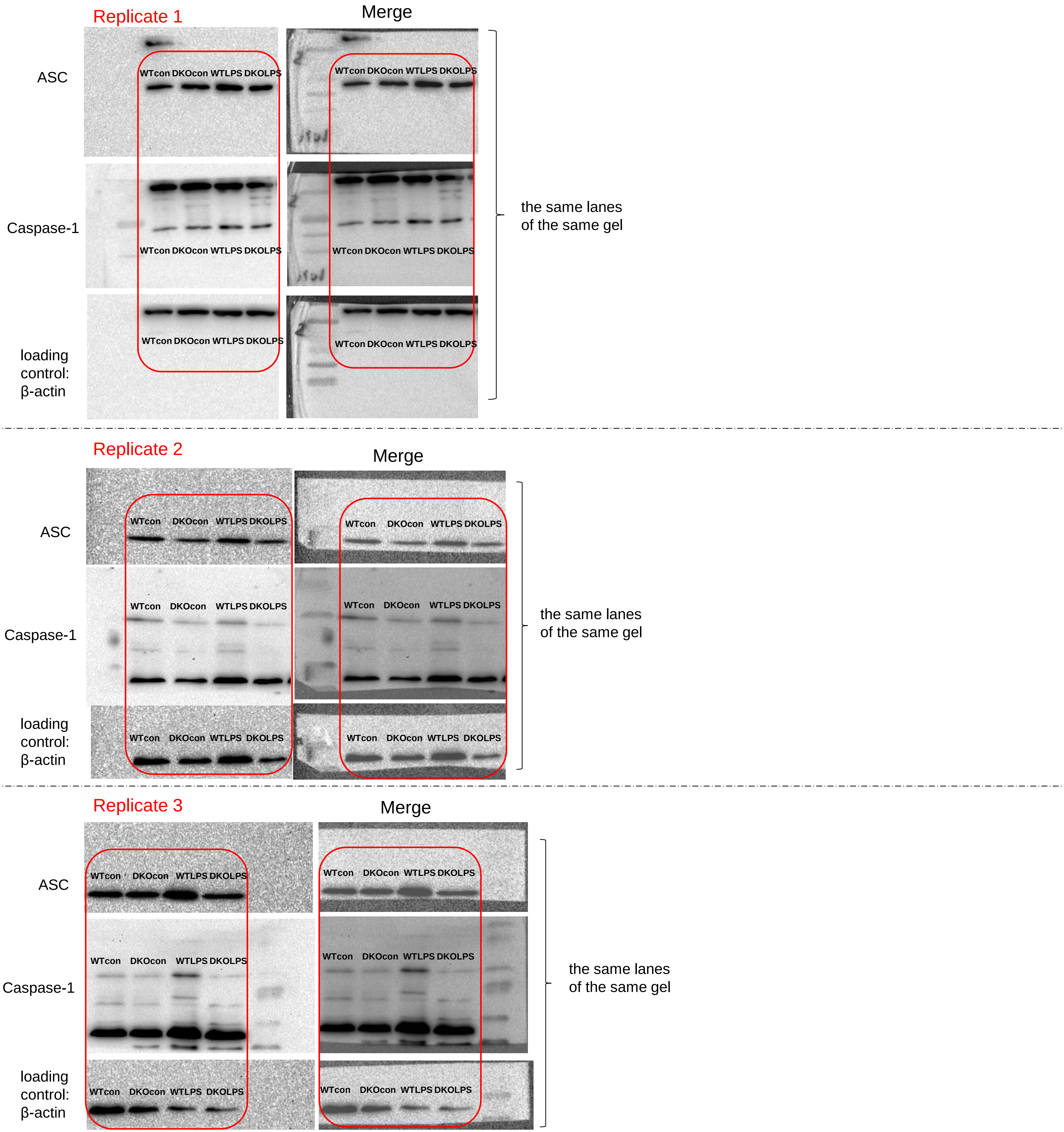

Full unedited blots of Figure 6D (For Ym1 protein)

Replicate 1

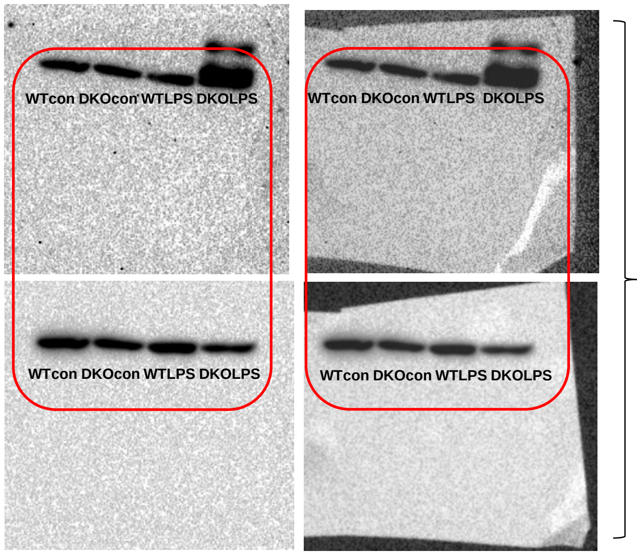

Replicate 2

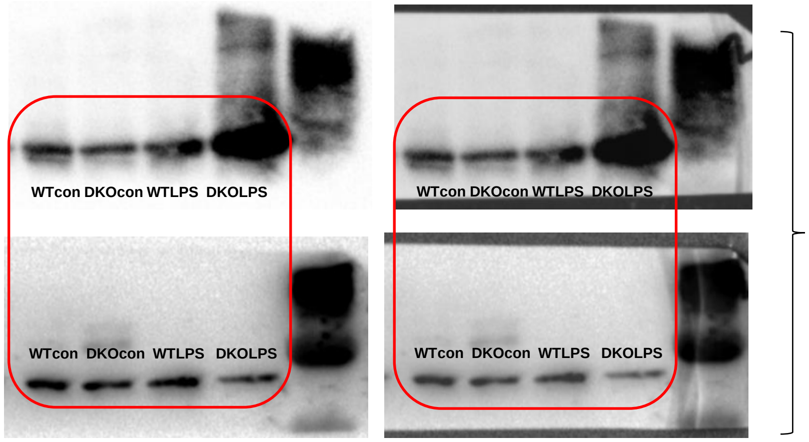

Replicate 3

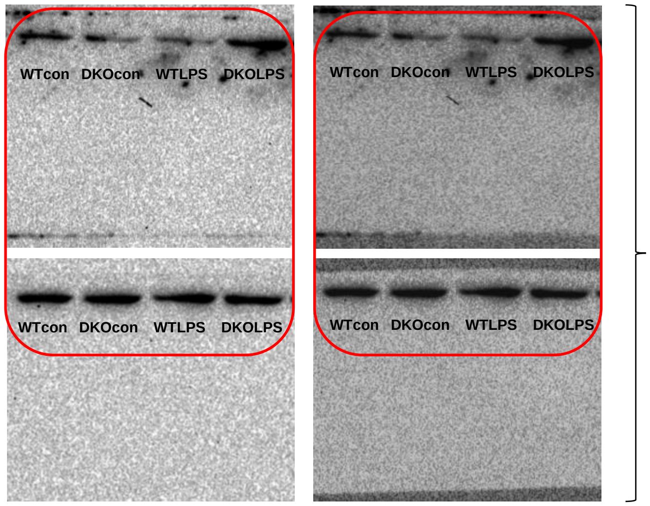

Full unedited blots of Figure 6D (For iNOS protein)

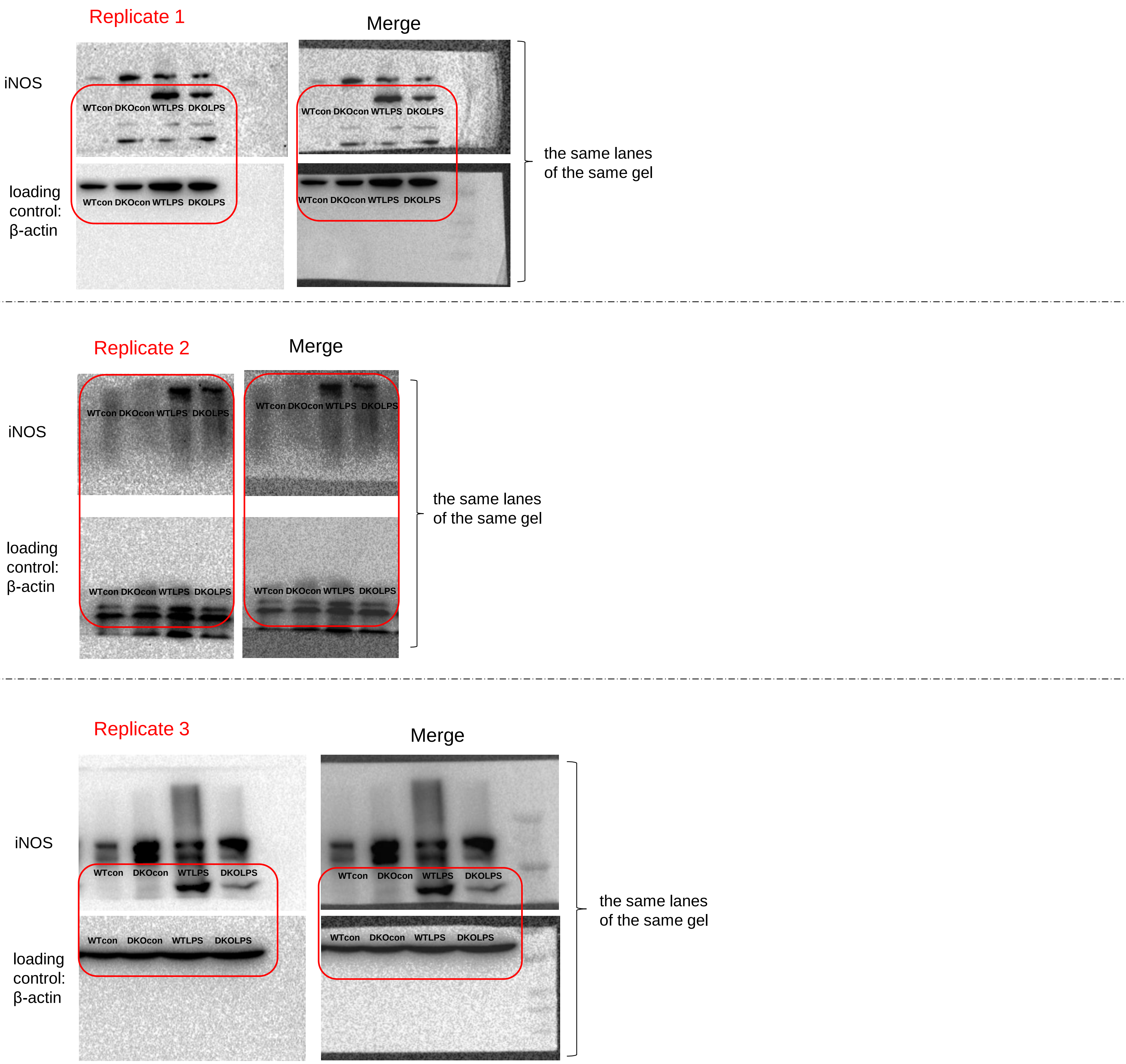

Full unedited blots of Figure 7A (For Ym1 protein)

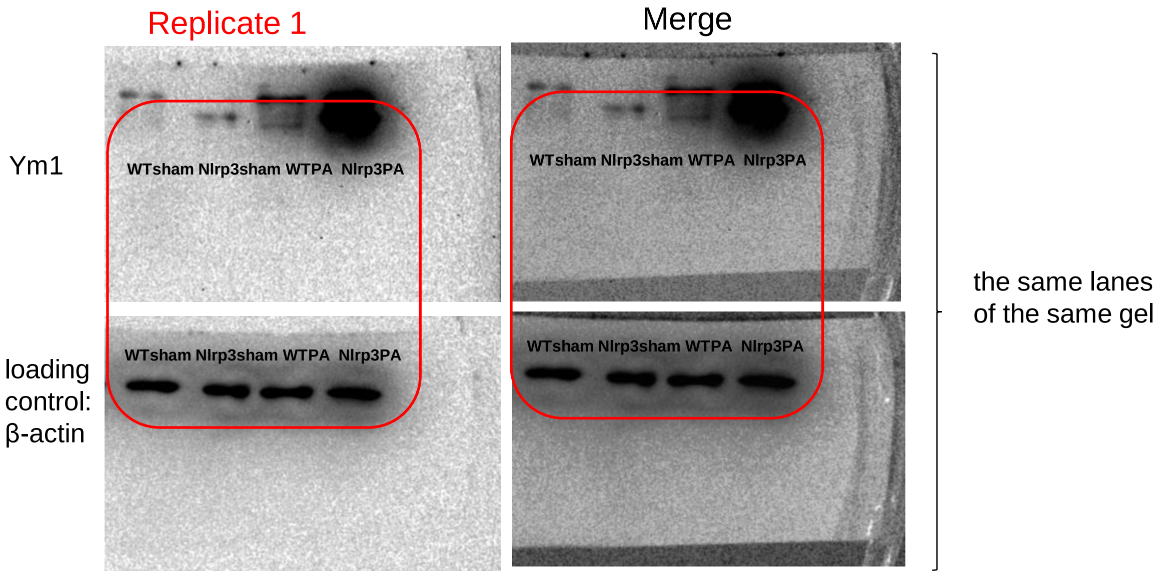

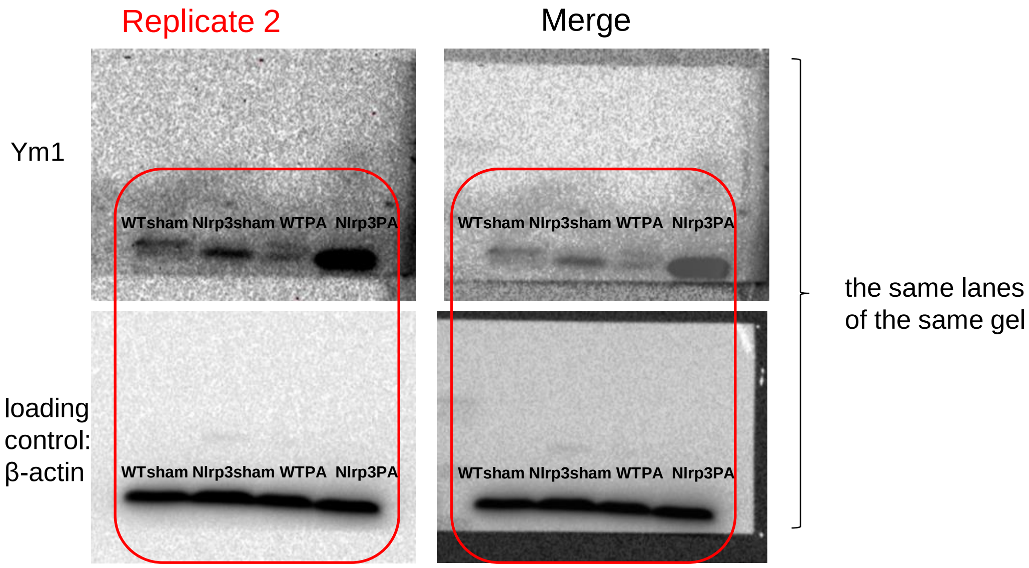

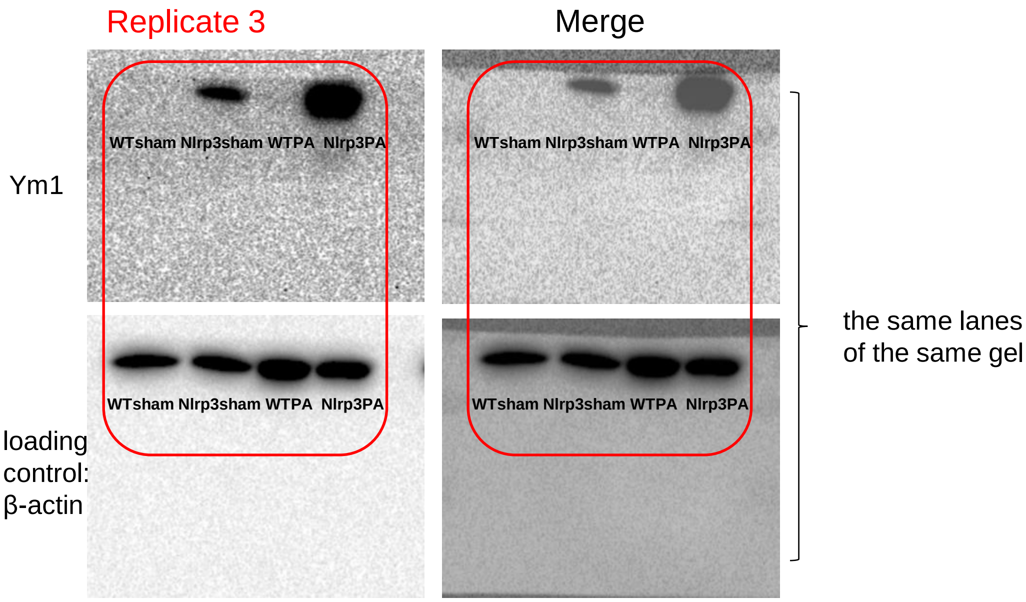

Full unedited blots of Figure 7B (For Ym1 protein)

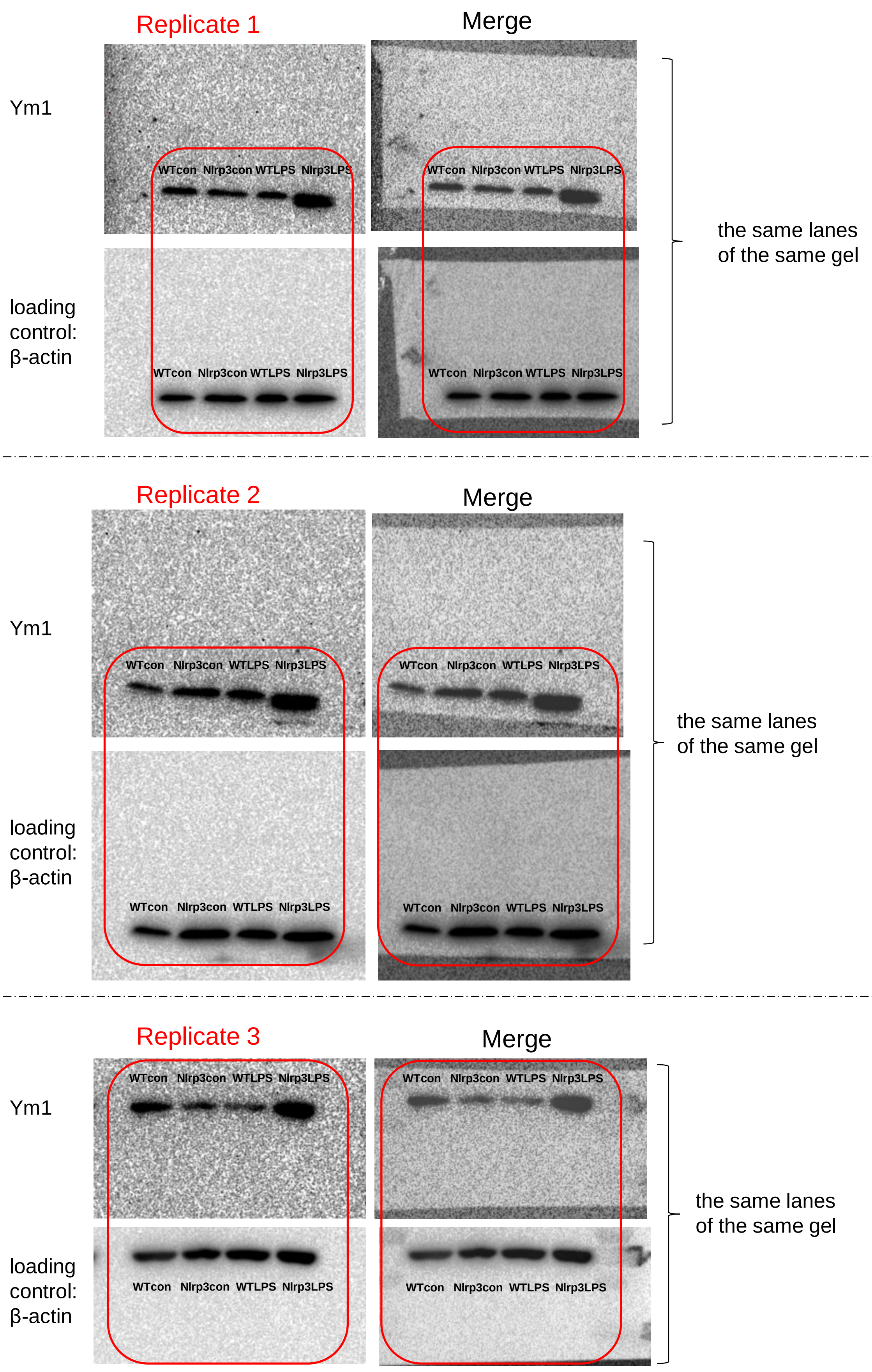

Full unedited blots of supplemental Figure S1D (For PADI2 & PADI4 proteins)

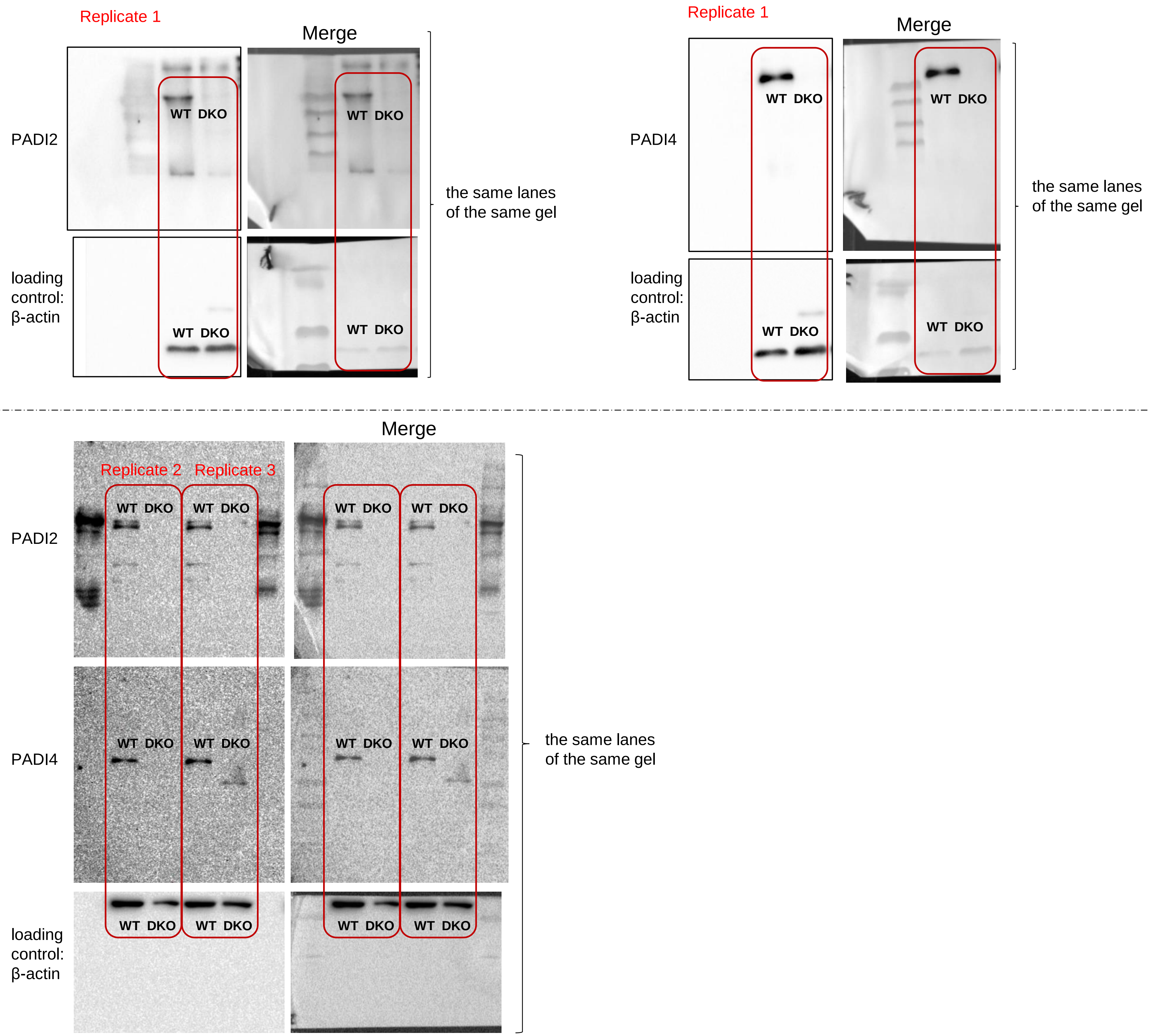

Full unedited blots of supplemental Figure S4B (For Ym1 protein)

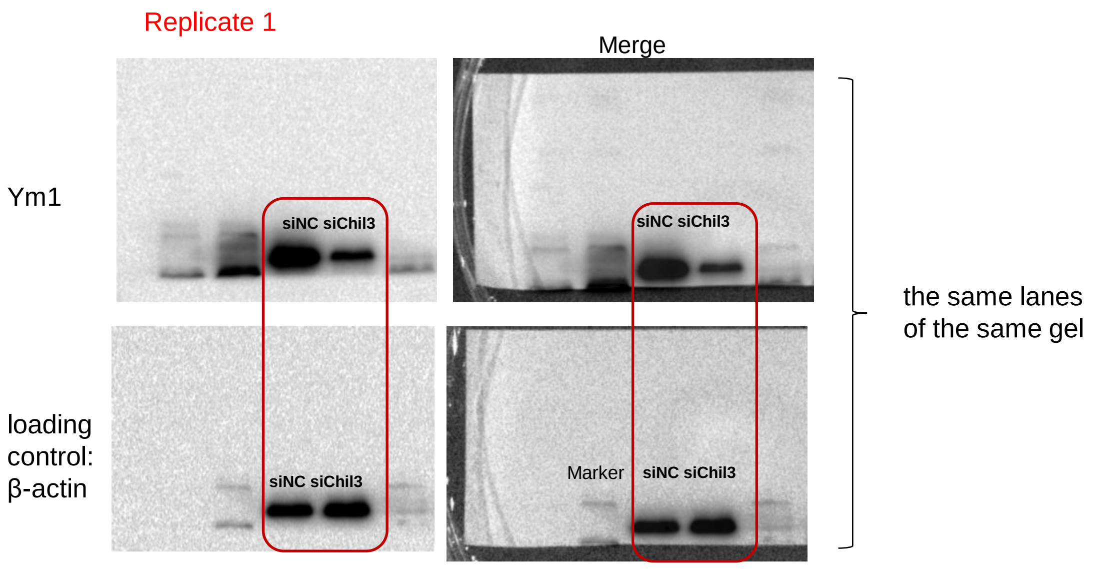

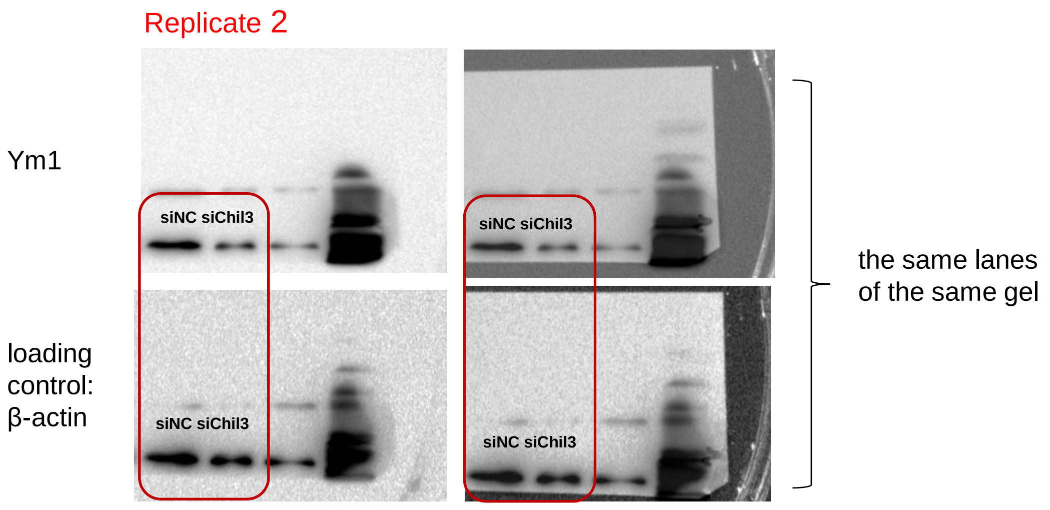

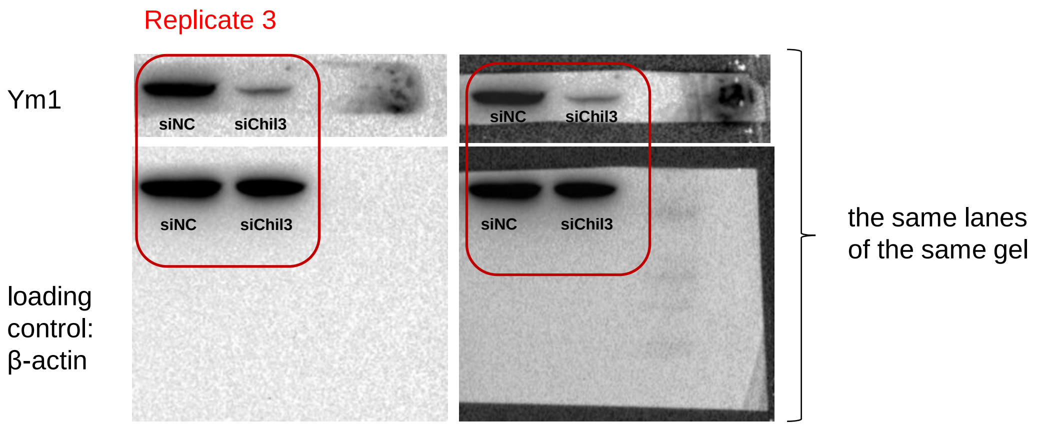

Supplement: Unedited blot and gel images [file jciinsight-9-181686-s211.pdf]
